# A Breast Cancer Stem Active Cobalt(III)‐Cyclam Complex Containing Flufenamic Acid with Immunogenic Potential

**DOI:** 10.1002/anie.202317940

**Published:** 2023-12-29

**Authors:** Jiaxin Fang, Owamagbe N. Orobator, Chibuzor Olelewe, Ginevra Passeri, Kuldip Singh, Samuel G. Awuah, Kogularamanan Suntharalingam

**Affiliations:** ^1^ School of Chemistry University of Leicester Leicester UK; ^2^ Department of Chemistry University of Kentucky Lexington KY USA; ^3^ Department of Pharmaceutical Sciences University of Kentucky Lexington KY USA

**Keywords:** Cancer Stem Cells, Cobalt, Damage-Associated Molecular Patterns, Immunotherapeutics, Non-Steroidal Anti-Inflammatory Drugs

## Abstract

The cytotoxic and immunogenic‐activating properties of a cobalt(III)‐cyclam complex bearing the non‐steroidal anti‐inflammatory drug, flufenamic acid is reported within the context of anti‐cancer stem cell (CSC) drug discovery. The cobalt(III)‐cyclam complex **1** displays sub‐micromolar potency towards breast CSCs grown in monolayers, 24‐fold and 31‐fold greater than salinomycin (an established anti‐breast CSC agent) and cisplatin (an anticancer metallopharmaceutical), respectively. Strikingly, the cobalt(III)‐cyclam complex **1** is 69‐fold and 50‐fold more potent than salinomycin and cisplatin towards three‐dimensionally cultured breast CSC mammospheres. Mechanistic studies reveal that **1** induces DNA damage, inhibits cyclooxygenase‐2 expression, and prompts caspase‐dependent apoptosis. Breast CSCs treated with **1** exhibit damage‐associated molecular patterns characteristic of immunogenic cell death and are phagocytosed by macrophages. As far as we are aware, **1** is the first cobalt complex of any oxidation state or geometry to display both cytotoxic and immunogenic‐activating effects on breast CSCs.

## Graphical abstract

Here we report the first investigation into the cytotoxic and immunogenic potential of a cobalt complex within the context of anti‐cancer stem cell (CSC) drug discovery. The cobalt complex is able to deliver flufenamic acid to CSCs, which perturbs the inhibitory damage‐associated molecular pattern axis and promotes immunogenic cell death. This subsequently leads to the phagocytosis of CSCs by macrophages.

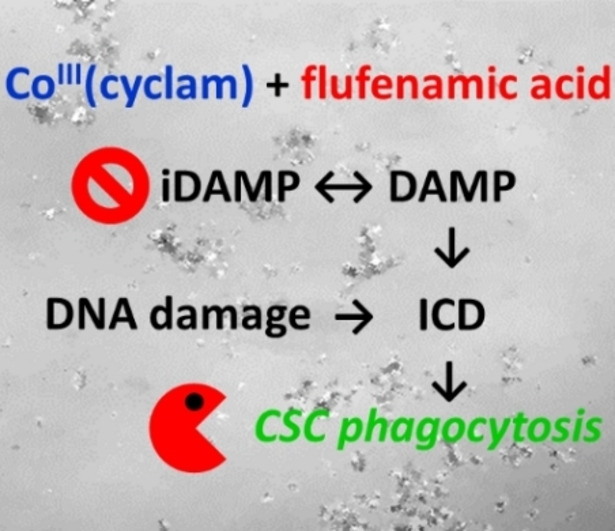

## Introduction

Cancer remains a leading cause of death worldwide, with nearly 10 million people dying from the disease in 2020 (which accounted for a staggering 1 in 6 of all recorded deaths for the year).^[1]^ The main cause of cancer related death is relapse and metastasis, both of which are linked to the presence of cancer stem cells (CSCs).^[2]^ CSCs are a sub‐population of tumour cells with stem cell‐like properties, including the ability to self‐renew and differentiate.^[3]^ CSCs also exhibit slower cell cycle profiles than bulk cancer cells, enabling them to evade traditional cancer treatments (chemotherapy and radiation) that rely on selectively killing fast growing cells.^[4]^ As CSCs tend to reside in hard‐to‐reach niches within the tumour microenvironment, they can also escape surgical interventions.^[5]^ Upon overcoming therapy, CSCs can reform tumours in the original site or promote colonisation of distant organs, often leading to multiple organ failure and fatal outcomes.^[6]^ Given the biological significance and clinical implications of CSCs there are multiple on‐going research programmes aimed at developing anticancer agents that can remove both bulk cancer cells and CSCs.^[7]^ Despite these endeavours there is currently no clinically approved agent (chemical or biologic) that can remove CSCs at clinically safe doses.

Most anti‐CSC agents currently under development target established CSC traits such as proteins associated with dysregulated signaling pathways, overactive organelles or cell surface markers.^[8]^ A relatively underexplored therapeutic target is components within the microenvironments in which CSCs reside.^[9]^ Certain CSCs are thought to exist in hypoxic regions within tumour niches.^[10]^ Hypoxia inducible factors (HIFs), HIF1α and HIF2α are thought to be vital factors in regulating and maintaining CSCs, and HIF1α has been shown to enhance CSC growth in three‐dimensional cultures under hypoxic conditions.^[11]^ Prodrugs capable of accumulating in the CSC microenvironment and undergoing activation could potentially kill CSCs effectively. Six‐coordinate cobalt(III) complexes with bioactive ligands can be used for this objective, given that the oxidised cobalt(III), d^6^ form is inert and the reduced cobalt(II), d^7^ form is relatively labile and can release coordinated bioactive ligands.^[12]^ There is a large body of work already reported on the development of cobalt(III) prodrugs capable of delivering therapeutic and imaging agents under hypoxic conditions, however studies focused on their anti‐CSC properties are rare.^[12]^

We have previously reported the anti‐CSC properties of cobalt(III)‐cyclam and cobalt(III)‐phenanthroline complexes appended to non‐steroidal anti‐inflammatory drugs (NSAIDs), namely naproxen, tolfenamic acid, and diflunisal.^[13]^ These cobalt(III) complexes were shown, through detailed biophysical studies, to release the attached NSAIDs under reducing conditions.^[13]^ NSAIDs inhibit cyclooxygenase‐2 (COX‐2), an enzyme that catalyzes the production of the inflammation mediator prostaglandin.^[14]^ COX‐2 is overexpressed in certain CSCs and implicated in their regulation.^[15]^ The release of NSAIDs from the cobalt(III)‐cyclam and cobalt(III)‐phenanthroline complexes thus sensitises CSCs (through the inhibition of COX‐2) to the cytotoxic reduced cobalt(II) form. Mechanistic studies indicated that the cobalt(III)‐cyclam and cobalt(III)‐phenanthroline complexes induce breast CSC apoptosis by genomic DNA damage and COX‐2 downregulation.^[13]^

Cancer cells that undergo immunogenic cell death (ICD) can stimulate immune cells to actively seek and destroy them by exposing or releasing damage‐associated molecular patterns (DAMPs).^[16]^ A recent study showed that prostaglandin acts as an inhibitory DAMP (iDAMP), thus preventing ICD of cancer cells and the corresponding downstream immunogenic effects.^[17]^ This study showed that the clinically used anticancer drug gemcitabine could be converted from a non‐ICD‐inducer into an ICD‐inducer by co‐administration with celecoxib, a COX‐2 inhibitor.^[17]^ CSCs that have undergone ICD can potentially act as vaccines and initiate a robust and durable immune response against CSCs. The activation of ICD in CSCs is an underexplored avenue in immunotherapy,^[18]^ and only a few metal complexes have been reported to induce ICD in CSCs (of any tissue type).^[19]^ The perturbation of iDAMPs by exogenous metal complexes to prompt ICD in CSCs has not been attempted to date.

**Scheme 1:**
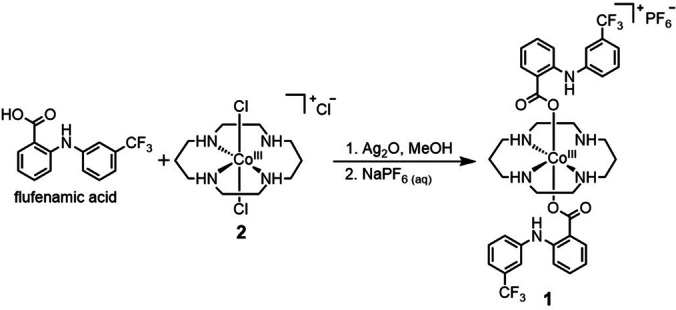
Reaction scheme for the preparation of cobalt(III)‐cyclam complex **1** from *trans*‐dichloro(cyclam)‐cobalt(III) chloride **2** and flufenamic acid.

Here, we have sought to expand the therapeutic scope of anti‐CSC cobalt(III) prodrugs by combining cobalt(III)‐cyclam with flufenamic acid, an NSAID that has been successfully used for chemoprevention in various cancer models.^[20]^ In addition to inhibiting COX‐2 and sensitizing CSCs to cytotoxic mechanisms, the flufenamic acid moiety is expected to enhance immunogenicity by facilitating DAMP exposure and release (through the downregulation of COX‐2). Specifically, we report the synthesis and characterisation of a cobalt(III)‐cyclam complex appended to two flufenamic acid units **1** (see Scheme 1 for chemical structure) and provide insight into its anti‐breast CSC potency (in monolayer and three‐dimensional cultures) and mechanism of action. The ability of **1** to inhibit COX‐2 expression and trigger DAMP release from breast CSCs is explored. The ability of **1** to promote breast CSC phagocytosis by macrophages is also investigated. To the best of our knowledge this is the first study to explore the cytotoxic and immunogenic potential of a cobalt coordination complex in the context of anti‐breast CSC therapeutics.

## Results and Discussion

The cobalt(III)‐cyclam complex attached to two flufenamic acid moieties **1** was prepared by reacting *trans*‐dichloro(cyclam)‐cobalt(III) chloride **2** ^[21]^ with two equivalence of flufenamic acid in methanol (dried over Na_2_SO_4_) with the added presence of excess silver(I) oxide (Scheme 1). The crude product was precipitated using diethyl ether prior to conversion to the corresponding hexafluorophosphate salt. The hexafluorophosphate salt was then subject to alumina column chromatography [dichloromethane: methanol (95 : 5)] which allowed the isolation of pure **1** as a tawny solid, in a reasonable yield (42 %). The cobalt(III) complex **1** was fully characterised by ^1^H, ^19^F{^1^H} and ^31^P{^1^H} NMR, infrared spectroscopy, high‐resolution ESI mass spectrometry, and elemental analysis (Figures S1–S7, see ESI). Attachment of flufenamic acid to cobalt(III)‐cyclam via the carboxylic acid group was confirmed by the disappearance of the hydroxyl signal (at 13.20 ppm in flufenamic acid, Figures S1 and S4) in the ^1^H NMR spectrum of **1**. Furthermore the aromatic proton signals shifted upfield relative to flufenamic acid, indicative of coordination to cobalt(III). The ATR‐FTIR spectrum for **1** displayed ν_asym_(CO_2_) and ν_sym_(CO_2_) stretching bands at 1580 cm^−1^ and 1329 cm^−1^, respectively (Figure S6). The difference, Δ, between the ν_asym_(CO_2_) and ν_sym_(CO_2_) stretching bands for **1** was 251 cm^−1^, suggestive of an unidentate coordination mode for the carboxylate group on flufenamic acid to the cobalt centre.^[22]^ A distinctive molecular ion peak with the appropriate isotopic pattern expected for **1** was observed in the high‐resolution ESI mass spectrum (*m/z*=819.2504 a.m.u, [**1**‐PF_6_]^+^), providing further evidence for product formation (Figure S7). The purity of **1** was confirmed by elemental analysis.

**Figure 1:**
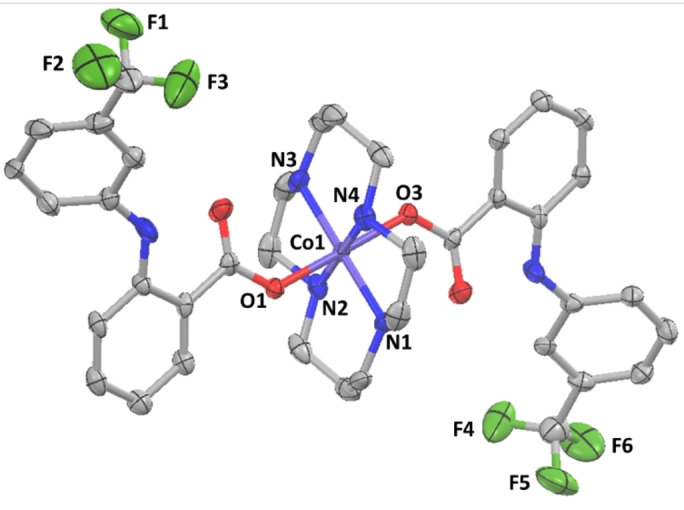
X‐ray crystal structure of **1**. Ellipsoids are set at 50 % probability level. Hydrogen atoms, the counter‐anion, and co‐crystallising solvent molecules have been omitted for clarity. Two unique molecules are present in the unit cell but only one is shown here for clarity.

Single crystals of **1** suitable for X‐ray diffraction studies were obtained by vapour diffusion of diethyl ether into the methanolic crude reaction mixture of **1** (CCDC 2287973, Figure 1, Table S1).^[23]^ Selected bond lengths and angles are presented in Table S2. The cationic component of **1** consists of a cobalt(III) centre with a distorted octahedral geometry (two unique molecules are present in the unit cell). The cobalt(III) centre is coordinated to cyclam via four nitrogen‐donor atoms and to two flufenamic acid moieties via the deprotonated hydroxyl moiety within the carboxylate group (Figure 1). The latter is consistent with the flufenamic acid‐to‐cobalt binding mode assignment obtained from the ATR‐FTIR spectroscopy studies (Figure S6). Within the CoN_4_ equatorial plane the average bond angle between nitrogen atoms *cis* to one another is 90.01° and the average axial O−Co−O bond angle is 179.25°, consistent with a distorted octahedral geometry. The average Co−N and Co−O bond lengths are consistent with the values reported for the only other octahedral cobalt(III)‐cyclam complex with carboxylate ligands resolved by X‐ray crystallography.^[13a]^

The lipophilicity of **1** was determined by measuring the extent to which it partitioned between octanol and water using the shake‐flask method. The LogP value for **1** was calculated to be 0.50±0.14, which is suggestive of amphiphilicity. The amphiphilic nature of **1** suggests that the cobalt(III) complex should be soluble in solutions used for cell studies and also be readily taken up by dividing cells. UV/Vis spectroscopy and ESI mass spectrometry studies were carried out to assess the stability of **1** in biologically relevant solutions. In DMSO, the absorption bands associated to **1** (50 μM) remained largely unchanged indicative of good stability (Figure S8). In H_2_O:DMSO (200 : 1) and PBS:DMSO (200 : 1) the UV/Vis trace associated to **1** (50 μM) decreased steadily over the course of 24 h at 37 °C (Figures S9 and S10). Nevertheless, the wavelengths associated to the π–π* and MLCT bands for **1** remained unaltered, suggestive of reasonable stability under these conditions (Figures S9 and S10). Upon addition of ascorbic acid or gluathione (0.5 mM, 10 equivalence) to **1** (50 μM) in H_2_O:DMSO (200 : 1), a marked change in the UV/Vis trace was observed followed by a decrease in absorption over the course of 24 h (Figures S11 and S12), yielding a spectrum similar to free flufenamic acid (Figure S13). This indicates that flufenamic acid is released from **1** in the presence of cellular reductants. The ESI negative mass spectrum of **1** (40 μM) in the presence of glutathione (0.4 mM, 10 equivalents) in H_2_O:DMSO (10 : 1) after 24 h incubation displayed a new peak corresponding to [flufenamic acid]^−^ (281 *m/z*) (Figure S14). Signals associated to cobalt(II)‐cyclam species were also detected in the corresponding ESI positive mass spectrum (Figure S15). This shows that flufenamic acid is released from **1**, presumably through reduction of the cobalt metal centre from Co(III) to Co(II).

The cytotoxicity of the cobalt(III) complex **1** towards bulk breast cancer cells (HMLER) and breast CSCs (HMLER‐shEcad) (grown in two‐dimensional cultures) was determined using the well‐established colorimetric MTT (3‐(4,5‐dimethylthiazol‐2‐yl)‐2,5‐diphenyltetrazolium bromide) assay. IC_50_ values were determined from dose‐response curves (Figure S16) and are presented in Table 1. The cobalt(III) complex **1** displayed sub‐micromolar toxicity towards breast CSCs and bulk breast cancer cells, with slightly higher potency towards breast CSCs. Notably, **1** exhibited 24‐fold and 31‐fold higher toxicity towards breast CSCs than salinomycin (a gold standard anti‐breast CSC agent) and cisplatin (a clinically used anticancer metallopharmaceutical), respectively (Table 1).^[24]^ Control studies showed that flufenamic acid displayed up to 171‐fold lower potency compared to **1** against HMLER or HMLER‐shEcad cells (Figure S17, Table 1), while *trans*‐dichloro(cyclam)‐cobalt(III) chloride **2** is non‐toxic towards HMLER or HMLER‐shEcad cells (IC_50_ value >100 μM).^[13a]^ When dosed as a 1 : 2 mixture, the combined treatment of **2** and flufenamic acid showed up to 89‐fold reduction in potency towards HMLER and HMLER‐shEcad cells compared to **1** (Figure S18, Table 1). This demonstrates that the preformed cobalt(III) complex **1** is significantly (*p* <0.05) better at killing bulk breast cancer cells and breast CSCs than a mixture of its individual components. To gauge therapeutic potential, the cytotoxicity of **1** towards embryonic kidney HEK 293 cells was determined. The cobalt(III) complex **1** was significantly less potent towards HEK 293 cells (IC_50_ value=2.49±0.36 μM, up to 14‐fold, *p*<0.05, Figure S19) than HMLER and HMLER‐shEcad cells, therefore **1** has the potential to potently kill bulk breast cancer cells and breast CSCs over non‐cancerous cells.

**Table 1:** IC_50_ values of the cobalt(III) complexes **1** and **2**, flufenamic acid, **2**+flufenamic acid (1 : 2), cisplatin, and salinomycin against HMLER cells, HMLER‐shEcad cells, and HMLER‐shEcad mammospheres.

Compound	HMLER IC_50_ [μM]^[a]^	HMLER‐shEcad IC_50_ [μM]^[a]^	Mammosphere IC_50_ [μM]^[b]^
**1**	0.27±0.03	0.18±0.003	0.27±0.02
**2** ^[c]^	>100	>100	>133
flufenamic acid	7.32±1.04	30.86±2.65	>133
**2**+flufenamic acid	20.01±0.68	15.96±0.57	n.d.
salinomycin^[c]^	11.43±0.42	4.23±0.35	18.50±1.50
cisplatin^[c]^	2.57±0.02	5.65±0.30	13.50±2.34

[a] Determined after 72 h incubation (mean of three independent experiments±SD). [b] Determined after 5 days incubation (mean of three independent experiments±SD). [c] Reported in references 24 and 26. n.d. not determined.

**Figure 2:**
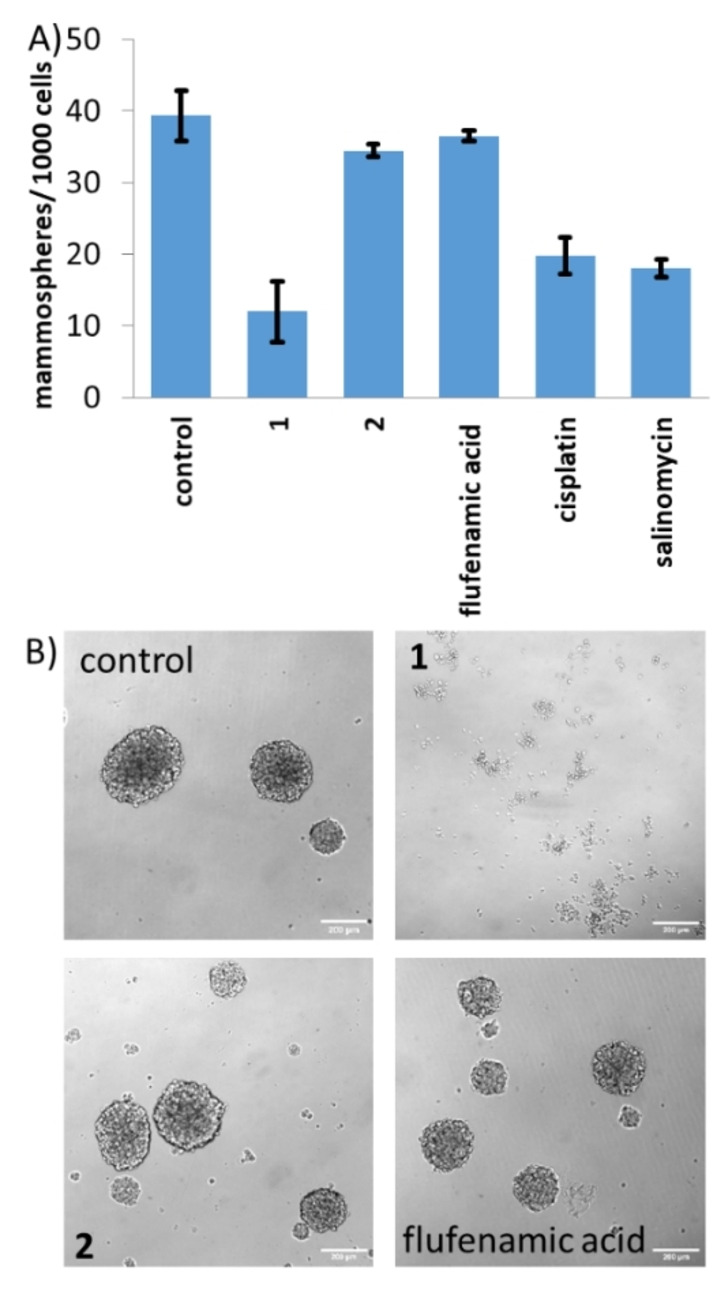
(A) Quantification of mammosphere formation with HMLER‐shEcad cells untreated and treated with **1**, **2**, flufenamic acid, salinomycin or cisplatin (at their IC_20_ values, 5 days). Error bars represent standard deviations. (B) Representative bright‐field images (×10) of HMLER‐shEcad mammospheres in the absence and presence of **1**, **2** or flufenamic acid (at their IC_20_ values, 5 days).

When breast CSCs are cultured in low attachment conditions without serum, three‐dimensional multicellular structures called mammospheres can be generated.^[25]^ The ability of a given compound to inhibit mammosphere formation or viability provides a reasonable indication of its translational potential. The aptitude of the cobalt(III) complex **1** to inhibit mammosphere formation was assessed using an inverted microscope. The addition of **1** (IC_20_ value for 5 days) to single cell suspensions of HMLER‐shEcad cells markedly reduced the number (by 69 %) and size of mammospheres formed (Figures 2A−B). Salinomycin and cisplatin (at their respective IC_20_ values for 5 days) displayed similar mammosphere inhibitory properties compared to **1** under identical conditions (Figures 2A–B and S20). Specifically, salinomycin and cisplatin reduced the number of mammospheres formed by 54 % and 50 %, respectively (Figure 2A). Treatment with *trans*‐dichloro(cyclam)‐cobalt(III) chloride **2** or flufenamic acid (at their respective IC_20_ value for 5 days) did not significantly change the number or size of mammospheres formed (Figures 2A–B). To establish the ability of the cobalt(III) complex **1** to reduce mammosphere viability, the colorimetric resazurin‐based reagent, TOX8 was used. The IC_50_ value (concentration required to reduce mammosphere viability by 50 %) of **1** was in the sub‐micromolar range, 69‐fold and 50‐fold lower than salinomycin and cisplatin, respectively (Figure S21, Table 1).^[26]^ Flufenamic acid or *trans*‐dichloro(cyclam)‐cobalt(III) chloride **2** did not display any observable mammosphere potency (IC_50_>133 μM, Figure S21).^[13a]^ Taken together the mammosphere studies show that **1** is effectively able to reduce mammosphere formation and viability, and that its capacity to do so is significantly greater than the current gold standard anti‐breast CSC agent, salinomycin.

**Figure 3:**
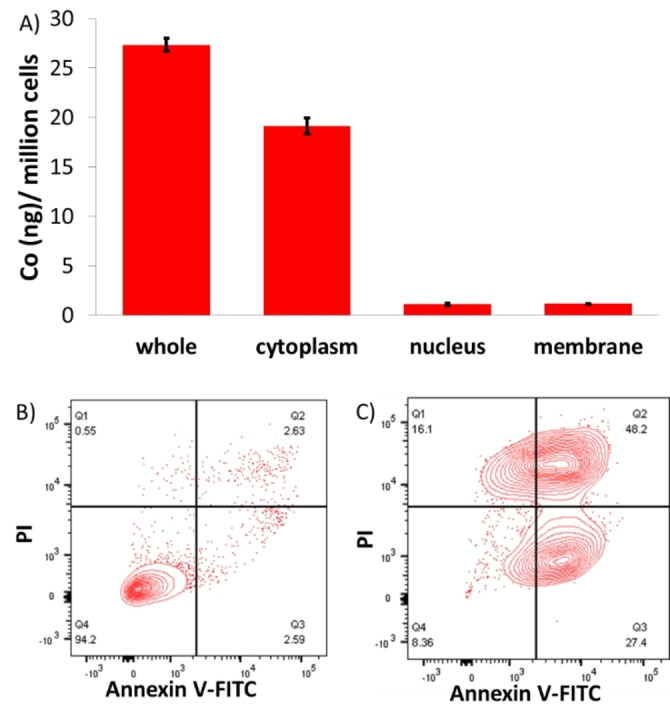
(A) Cobalt content in whole cell, cytoplasm, nucleus, and membrane fractions isolated from HMLER‐shEcad cells treated with **1** (0.5 μM for 24 h). Error bars represent standard deviations. (B–C) FITC Annexin V‐propidium iodide binding assay plots of untreated HMLER‐shEcad cells and HMLER‐shEcad cells treated with **1** (4×IC_50_ value for 72 h).

Having shown that the cobalt(III) complex **1** is able to potently kill breast CSCs grown in monolayers and three‐dimensional cultures, further cell‐based studies were conducted to shed light on its mechanism of action. Cellular uptake and fractionation studies were performed to determine the breast CSC internalisation and intracellular localisation of **1**. HMLER‐shEcad cells were incubated with **1** (0.5 μM for 24 h) and the cobalt content was measured in the whole cell, cytoplasmic, nuclear, and membrane fractions by inductively coupled plasma mass spectrometry (ICP‐MS). A substantial amount of **1** was internalised into breast CSCs (27.37 ng of Co/ million cells) taking into account the administration dose (Figure 3A). A large amount of internalised **1** was detected in the cytoplasm and appreciable amounts of **1** were also found in the membrane and nuclear fractions (Figure 3A). The latter is consistent with the presence of the flufenamic acid moiety in **1**, which is known to target COX‐2 localised on the nuclear envelope.^[27]^ The distribution of **1** inside breast CSCs suggests that **1**‐induced breast CSC toxicity could be related to interactions with cytoplasmic biomolecules, however, a genomic DNA‐dependent mechanism could also be possible. To determine if **1** can interact with cytoplasmic biomolecules, we carried out time course ESI mass spectrometry studies with **1** in the presence of histidine, cysteine, and glucose (biomolecules present in millimolar quantities in the cytoplasm), and moreover, in the presence of the cytoplasmic extract of HMLER‐shEcad cells. Specifically, **1** (80 μM) was incubated with histidine (0.8 mM), cysteine (0.8 mM), glucose (0.8 mM) or the cytoplasmic extract of HMLER‐shEcad cells (0.5 million cells) in H_2_O:DMSO (10 : 1) at 37 °C and the ESI mass spectra were obtained immediately and after 24 h. Under all of the conditions tested a clear signal with the expected isotopic pattern corresponding to intact **1** was observed (Figures S22–S23), indicative of little or no interaction between **1** and histidine, cysteine, glucose, or any components within the cytoplasmic extract of HMLER‐shEcad cells. This implies that **1**‐induced breast CSC toxicity is not related to interactions with cytoplasmic biomolecules.

Given that **1** was able to enter the nucleus and similar cobalt(III)‐cyclam complexes are known to damage genomic DNA, immunoblotting studies were conducted to assess the ability of **1** to induce genomic DNA damage in breast CSCs. HMLER‐shEcad cells incubated with **1** (0.2–0.8 μM for 24 h) displayed a noticeable increase in the expression of phosphorylated H2AX (γH2AX) and phosphorylated CHK2, indicative of DNA damage (Figure S24A).^[28]^ When DNA lesions induced by exogenous agents are so severe that they cannot be repaired, apoptosis can result.^[29]^ HMLER‐shEcad cells treated with **1** (0.1–0.4 μM for 72 h) displayed a visible increase in the expression of cleaved caspase 3, cleaved caspase 7, and cleaved PARP‐1 compared to untreated control cells, indicative of caspase‐dependent apoptosis (Figure S24B).^[30]^ Apoptosis results in changes to cell morphology including the rearrangement of the cell membrane. During this process, phosphatidylserine residues are translocated from the membrane interior to the membrane exterior, which can be detected by Annexin V.^[31]^ Compromised cell membrane architectures also promote propidium iodide uptake. Using the FITC Annexin V‐propidium iodide flow cytometry assay, we determined if the effect of **1** on the cell membrane of breast CSCs was consistent with apoptosis. Treatment of HMLER‐shEcad cells with **1** (2×IC_50_ value and 4×IC_50_ value for 72 h) induced a sizeable population of cells to expose phosphatidylserine on the cell membrane exterior and take up propidium iodide, indicative of early‐ and late‐stage apoptosis (Figures 3B–C and S25). This was comparable to treatment with cisplatin (25 μM for 72 h), an established apoptosis inducer (Figure S25). Taken together, the immunoblotting and flow cytometric studies show that **1** induces genomic DNA damage and caspase‐dependent apoptotic breast CSC death.

As the cobalt(III) complex **1** is able to release flufenamic acid under reducing conditions (Figures S11–15), flow cytometric studies were conducted to determine if **1** could downregulate COX‐2. HMLER‐shEcad cells pre‐treated with lipopolysaccharide (LPS) (2.5 μM for 24 h), to increase basal COX‐2 levels (Figure S26), were treated with **1** (IC_50_ value or 2×IC_50_ value for 48 h) and flufenamic acid (20 and 40 μM for 48 h), and the COX‐2 expression was determined by flow cytometry. COX‐2 expression decreased significantly upon treatment with **1** (Figure S27A). A similar effect was observed for flufenamic acid (Figure S27B). This shows that **1** is able to downregulate COX‐2 expression in HMLER‐shEcad cells. To determine if **1** induces COX‐2‐dependent breast CSC death, cytotoxicity studies were performed with HMLER‐shEcad cells pre‐treated with LPS (2.5 μM for 24 h). The potency of **1** towards HMLER‐shEcad cells decreased significantly (IC_50_ value=0.49±0.09 μM, 2.7‐fold, *p*<0.05) under these conditions (Figure S28). Collectively, the flow cytometric and cytotoxicity studies suggest that the mechanism of action of **1** could be related to COX‐2 downregulation.

**Figure 4:**
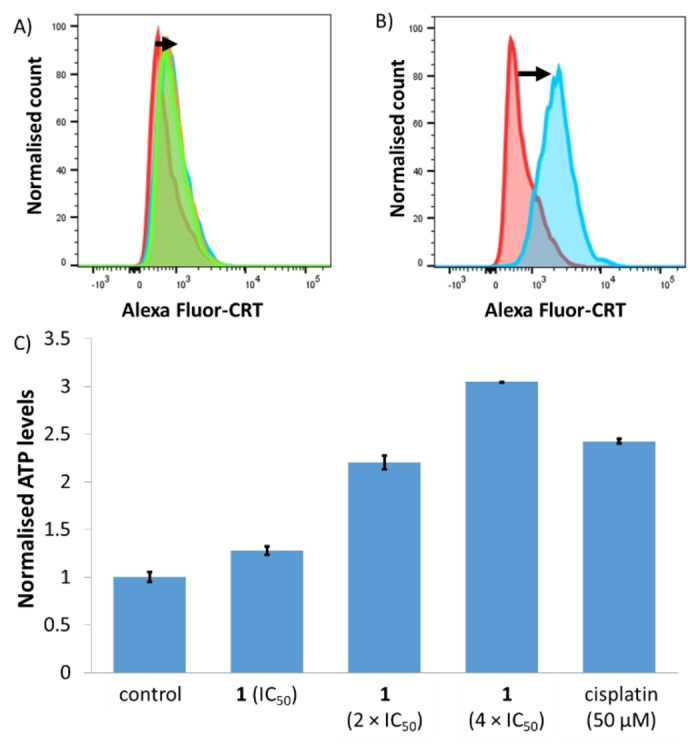
(A) Representative histograms displaying the green fluorescence emitted by anti‐CRT Alexa Fluor 488 nm antibody‐stained HMLER‐shEcad cells untreated (red), and treated with **1** (IC_50_ value for 24 h) (blue) or **1** (2×IC_50_ value for 24 h) (orange) or **1** (4×IC_50_ value for 24 h) (green). (B) Representative histograms displaying the green fluorescence emitted by anti‐CRT Alexa Fluor 488 nm antibody‐stained HMLER‐shEcad cells untreated (red), and treated with cisplatin (150 μM for 24 h) and thapsigargin (7 μM for 24 h) (blue). (C) Normalised extracellular ATP released from HMLER‐shEcad cells untreated and treated with **1** (IC_50_ value, 2×IC_50_ value, and 4×IC_50_ value for 24 h) or cisplatin (50 μM for 24 h). Error bars represent standard deviations.

COX‐2/prostaglandin E2 blockade was recently shown to be an effective strategy to promote ICD in bulk cancer cells.^[17]^ This approach could be translated to CSCs. Given that the cobalt(III) complex **1** downregulates COX‐2 in breast CSCs, we investigated whether this property could encourage DAMPs release from breast CSCs. The three major DAMPs associated with ICD in cancer cells are calreticulin (CRT), adenosine triphosphate (ATP), and nuclear high mobility group box 1 (HMGB‐1).^[16b]^ During the early stages of apoptosis, CRT is translocated from the endoplasmic reticulum to the cell membrane and acts as a signal to assist phagocytosis by immune cells.^[32]^ HMLER‐shEcad cells incubated with **1** (IC_50_ value, 2×IC_50_ value or 4×IC_50_ value for 24 h) exhibited appreciably higher levels of CRT on their cell membrane than untreated control cells (Figure 4A). HMLER‐shEcad cells treated with cisplatin (150 μM for 24 h) and thapsigargin (7 μM for 24 h) also displayed a marked increase in CRT on their cell membrane (Figure 4B). ATP is released from dying cells during the blebbing phase of apoptosis and acts as a signal for immune cells to locate dying cells (prior to engulfment).^[16a]^ The release of ATP from HMLER‐shEcad cells treated with **1** (IC_50_ value, 2×IC_50_ value or 4×IC_50_ value for 24 h) was determined by measuring the amount of ATP in the supernatant using a luciferase‐based assay. HMLER‐shEcad cells dosed with **1** released ATP in a dose‐dependent manner (up to 3‐fold more than untreated cells) (Figure 4C). Treatment of HMLER‐shEcad cells with cisplatin (50 μM for 24 h) also induced a significant amount of ATP to be released (2.4‐fold more than untreated cells) (Figure 4C). Another feature of ICD is the release of HMGB‐1 upon plasma membrane permeabilization.^[33]^ HMGB‐1 enables antigen processing and presentation to T‐cells.^[33]^ Immunoblotting studies showed that the relative amount of HMGB‐1 in HMLER‐shEcad cells remained somewhat unchanged upon treatment with **1** at sub‐micromolar concentrations (0.1–0.4 μM) after a relatively long exposure time (72 h) (Figure S29A). Contrastingly, HMGB‐1 in HMLER‐shEcad cells reduced significantly upon treatment with **1** at micromolar concentrations (2–10 μM for 24 h) after a shorter exposure time (24 h) (Figure S29B). Collectively, this suggests that HMGB‐1 is effectively released under certain conditions. Overall, the DAMP detection studies show that **1** promotes CRT cell surface exposure and ATP and HMGB‐1 release, and hence this indicates that the mode of cell death induced by **1** is consistent with ICD.

To provide some insight into the structure‐function relationship of cobalt(III)‐cyclam complexes with respect to ICD induction, we probed the ability of the previously reported cobalt(III)‐cyclam complex bearing two naproxen moieties **3** (see Figure S30 for chemical structure) to induce ICD of breast CSCs. According to the luciferin‐based ATP assay, HMLER‐shEcad cells dosed with **3** (IC_50_ value, 2×IC_50_ value or 4×IC_50_ value for 24 h) released ATP in a dose‐dependent manner (up to 2.2‐fold more than untreated cells) (Figure S31), similar to HMLER‐shEcad cells dosed with **1** (Figure 4C). Flow cytometry studies showed that HMLER‐shEcad cells incubated with **3** (IC_50_ value, 2×IC_50_ value or 4×IC_50_ value for 24 h) did not noticeably increase CRT exposure on their cell membrane compared to untreated control cells (Figure S32). Immunoblotting studies showed that the relative amount of HMGB‐1 in HMLER‐shEcad cells remained unchanged upon treatment with **3** at micromolar concentrations (2–10 μM for 24 h) suggesting that HMGB‐1 was not effectively released (Figure S33). The DAMP detection studies show that **3** promotes ATP release but not HMGB‐1 release or CRT exposure, and therefore the mode of cell death induced by **3** is not fully consistent with ICD. Taking the DAMP detection studies of cobalt(III)‐cyclam complexes **1** and **3** collectively, it is evident that the NSAID component is a determining factor in the ability of the cobalt(III)‐cyclam complexes to induce ICD of breast CSCs.

**Figure 5:**
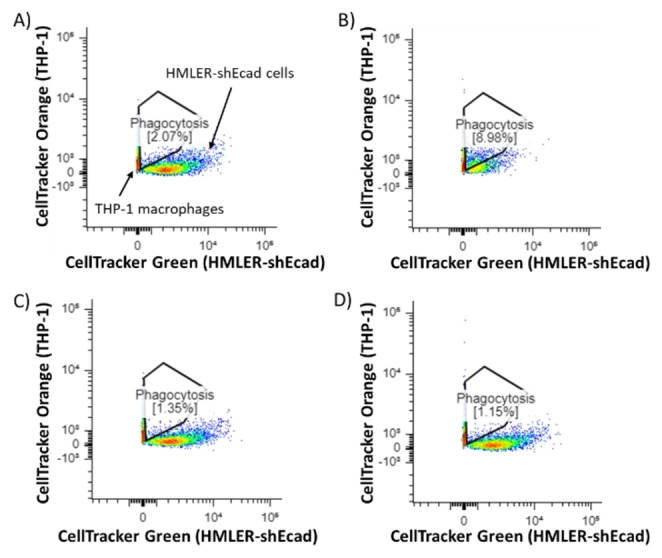
Representative two‐dimensional scatter plots of CellTracker Green‐stained HMLER‐shEcad cells (A) untreated and treated with (B) **1** (5 μM, 24 h) or (C) flufenamic acid (20 μM, 24 h) or (D) **2** (20 μM, 24 h), and then co‐cultured with CellTracker Orange‐stained THP‐1 macrophages for 2 h. The population of HMLER‐shEcad cells phagocytosed by THP‐1 macrophages is indicated.

As the cobalt(III)‐cyclam complex **1** was able to induce the hallmarks of ICD in breast CSCs, we determined the ability of breast CSCs treated with **1** to be engulfed by macrophages using an in vitro phagocytosis assay. Specifically, HMLER‐shEcad cells pre‐stained with CellTracker Green were treated with **1** (5 μM for 24 h) and then incubated with macrophages pre‐stained with CellTracker Orange for 2 h. The macrophages were obtained by differentiating acute monocytic leukaemia THP‐1 cells with phorbol 12‐myristate 13‐acetate (100 nM for 72 h). Phagocytosis, identified by the overlap of HMLER‐shEcad cells with macrophages, was measured quantitatively by flow cytometry. The population of double‐stained macrophages and engulfed HMLER‐shEcad cells is indicated in the two‐dimensional scatter plots shown in Figure 5. This analysis showed that **1** (5 μM for 24 h) was able to significantly enhance phagocytosis of CellTracker Green‐stained HMLER‐shEcad cells by CellTracker Orange‐stained macrophages (4.3‐fold increase compared to untreated cells, Figure 5). CellTracker Green‐stained HMLER‐shEcad cells treated with **2** (20 μM for 24 h) or flufenamic acid (20 μM for 24 h) did not prompt phagocytosis by CellTracker Orange‐stained macrophages (Figure 5). This shows that only the preformed cobalt(III) complex **1**, and not its individual components, is able to induce phagocytosis of breast CSCs by macrophages. As expected, cisplatin 150 μM for 24 h) and thapsigargin (7 μM for 24 h) markedly enhanced phagocytosis of CellTracker Green‐stained HMLER‐shEcad cells by CellTracker Orange‐stained macrophages (Figure S34). Overall, these results show that the cobalt(III)‐cyclam complex **1** is able to kill breast CSCs in a manner that promotes engulfment by macrophages.

**Figure 6:**
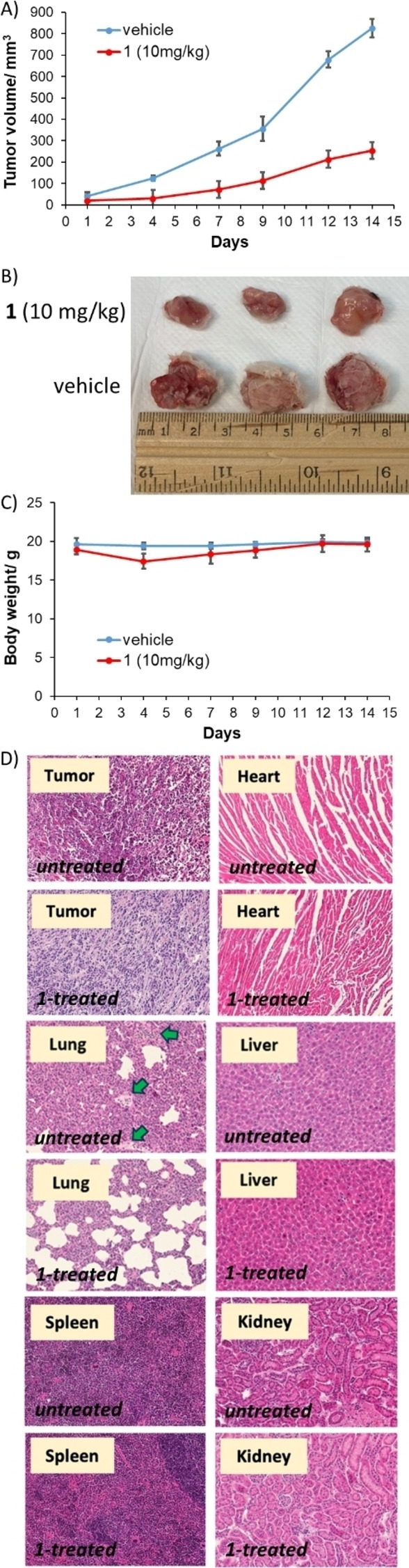
(A) The change in tumour volume of 4T1‐bearing mice (1 million cells inoculated, *n*=5) over 14 days, following intraperitoneal administration of **1** or the vehicle three times a week. Error bars represent standard deviations. (B) Representative images of excised tumours from **1**‐ or vehicle‐treated mice. (C) The change in body weight of **1**‐ or vehicle‐treated mice during the efficacy study lasting 14 days. Error bars represent standard deviations. (D) Hematoxylin and eosin staining of tumour, lungs, heart, liver, spleen, and kidney tissue obtained from **1**‐treated mice and vehicle‐treated mice. Metastatic sites in the lung control sample are shown with green arrows.

The in vivo antitumour efficacy of **1** was evaluated in an immunocompetent 4T1 metastatic triple‐negative breast cancer mouse model. The cobalt(III)‐cyclam complex **1** was administered intraperitoneally three times a week at a dose of 10 mg kg^−1^ (*n*=5 mice). An independent control group (*n*=5 mice) was treated with the vehicle at the same time points via the same administration route. As depicted in Figure 6A–B, tumour growth was significantly inhibited in the **1**‐treated group with respect to the control group. The **1**‐treated group also maintained their body weight (Figure 6C) relative to the control group throughout the course of the study. Histological studies showed reduced cellularity in tumour tissue obtained from the **1**‐treated group compared to the control group indicative of promising in vivo potency (Figure 6D). Further, the **1**‐treated group displayed reduced tumour colonisation of lung tissue compared to the control group, implying that **1** is able to prevent metastasis of breast cancer cells to the lungs (Figure 6D). There was no significant difference between the cellularity of the treated and control groups for the heart, liver, spleen or kidney samples, demonstrating a good safety profile for **1** in vivo (Figure 6D). Overall, the in vivo studies clearly show that **1** is able to effectively reduce breast tumour growth in a murine model and inhibit metastasis to the lungs without inducing significant systemic toxicity.

## Conclusion

In summary we report the synthesis, characterisation, and anti‐breast CSC properties of a cobalt(III)‐cyclam complex **1** comprising of two flufenamic acid moieties. Single crystal X‐ray crystallography studies showed that **1** adopts a distorted octahedral structure, with cyclam occupying the equatorial core and flufenamic acid in the axial positions (coordinated to cobalt via a single oxygen atom within the carboxylate group). UV/Vis spectroscopy and ESI mass spectrometry studies suggested that **1** is able to release flufenamic acid under reducing conditions. Cytotoxicity studies showed that **1** displayed sub‐micromolar potency towards bulk breast cancer cells and breast CSCs grown in monolayer cultures. Notably, this was 24‐fold and 31‐fold higher than salinomycin and cisplatin, respectively. The cobalt(III)‐cyclam complex **1** was also able to inhibit the formation and viability of three‐dimensional mammospheres within the sub‐micromolar range. The inhibitory and cytotoxic effect of **1** on mammospheres was superior than the effect by salinomycin and cisplatin under identical conditions. Mechanistic studies showed that **1** is able to readily enter breast CSCs, access the nucleus, and induce genomic DNA damage. The latter is then thought to prompt caspase‐dependent apoptosis. Furthermore, **1** significantly reduced COX‐2 expression in breast CSCs, thanks to the presence of two flufenamic acid moieties. Inspired by a recent report that linked COX‐2 inhibition (or reduction in prostaglandin E2) to ICD activation,^[17]^ the ability of **1** to evoke DAMPs in breast CSCs was investigated. DAMP detection studies showed that breast CSCs treated with **1** exhibited higher levels of CRT on their cell surface than untreated breast CSCs. The cobalt(III)‐cyclam complex **1** also promoted extracellular ATP and HMGB‐1 release suggesting that the mode of cell death evoked by **1** is consistent with ICD. Phagocytosis studies showed that breast CSCs treated with **1** were effectively engulfed by macrophages, highlighting the promising immunogenic potential of **1**. As far as we know, this is the first investigation into the cytotoxic and immunogenic potential of a cobalt complex within the context of anti‐breast CSC therapy. This study reinforces the therapeutic potential of cobalt complexes and paves the way for the development of other metal‐NSAID complexes as immunotherapeutics for CSC‐focused therapy.

## Conflict of interest

S.G.A. serves on the advisory board and is Chief Science Officer for Phronesis AI.

1

## Supporting information

As a service to our authors and readers, this journal provides supporting information supplied by the authors. Such materials are peer reviewed and may be re‐organized for online delivery, but are not copy‐edited or typeset. Technical support issues arising from supporting information (other than missing files) should be addressed to the authors.

Supporting Information

Supporting Information

## Data Availability

The data that support the findings of this study are available from the corresponding author upon reasonable request.
