# Supplementary material for: A Breast Cancer Stem Active Cobalt(III)‐Cyclam Complex Containing Flufenamic Acid with Immunogenic Potential
Source: Angew Chem Int Ed Engl. 2023 Dec 29;63(6):e202317940. doi: 10.1002/anie.202317940 (PMC10952489; doi:10.1002/anie.202317940)
Supplement: Supplementary file 2 — Supporting Information [file ANIE-63-0-s001.pdf]

Angewandte Chemie
Eine Zeitschrift der Gesellschaft Deutscher Chemiker

Supporting Information

A Breast Cancer Stem Active Cobalt(III)-Cyclam Complex Containing Flufenamic Acid with Immunogenic Potential

J. Fang, O. N. Orobator, C. Olelewe, G. Passeri, K. Singh, S. G. Awuah, K. Suntharalingam**

Supporting Information

Table of Content

Experimental Details

Figure S1. ^{1}H NMR spectrum of **1** in DMSO-d_6.
Figure S2. $^{19}F\{^{1}H\}$ NMR spectrum of **1** in DMSO-d_6.
Figure S3. $^{31}P\{^{1}H\}$ NMR spectrum of **1** in DMSO-d_6.
Figure S4. ^{1}H NMR spectrum of flufenamic acid in DMSO-d_6.
Figure S5. $^{19}F\{^{1}H\}$ NMR spectrum of flufenamic acid in DMSO-d_6.
Figure S6. ATR-FTIR spectra of **1** in the solid form.
Figure S7. High resolution ESI mass spectrum (positive mode) of **1**.
Table S1. Crystallographic data for cobalt(III) complex **1**.
Table S2. Selected bond lengths (Å) and angles (°) for cobalt(III) complex **1**.
Figure S8. UV-Vis spectrum of **1** (50 μM) in DMSO over the course of 24 h at 37 ^{o}C.
Figure S9. UV-Vis spectrum of **1** (50 μM) in H_2O:DMSO (200:1) over the course of 24 h at 37 ^{o}C.
Figure S10. UV-Vis spectrum of **1** (50 μM) in PBS:DMSO (200:1) over the course of 24 h at 37 ^{o}C.
Figure S11. UV-Vis spectrum of **1** (50 μM) in the presence of ascorbic acid (500 μM) in H_2O:DMSO (200:1) over the course of 24 h at 37 ^{o}C.
Figure S12. UV-Vis spectrum of **1** (50 μM) in the presence of glutathione (500 μM) in H_2O:DMSO (200:1) over the course of 24 h at 37 ^{o}C.

Figure S13. UV-Vis spectrum of flufenamic acid (50 μM) in H_2O:DMSO (200:1) at 37 ^oC.

Figure S14. ESI mass spectrum (negative mode) of **1** (40 μM) in H_2O:DMSO (10:1), in the presence of glutathione (400 μM) after 24 h.

Figure S15. ESI mass spectrum (positive mode) of **1** (40 μM) in H_2O:DMSO (10:1), in the presence of glutathione (400 μM) after 24 h.

Figure S16. Representative dose-response curves for the treatment of HMLER and HMLER-shEcad cells with **1** after 72 h incubation. Error bars represent standard deviations.

Figure S17. Representative dose-response curves for the treatment of HMLER and HMLER-shEcad cells with flufenamic acid after 72 h incubation. Error bars represent standard deviations.

Figure S18. Representative dose-response curves for the treatment of HMLER and HMLER-shEcad cells with a mixture of **2** and flufenamic acid (1:2) after 72 h incubation. Error bars represent standard deviations.

Figure S19. Representative dose-response curves for the treatment of HEK 293 cells with **1** after 72 h incubation. Error bars represent standard deviations.

Figure S20. Representative bright-field images (× 10) of HMLER-shEcad mammospheres in the absence and presence of salinomycin or cisplatin at their IC_{20} values (5 days incubation).

Figure S21. Representative dose-response curves for the treatment of HMLER-shEcad mammospheres with **1** or flufenamic acid after 5 days incubation. Error bars represent standard deviations.

Figure S22. ESI mass spectrum (positive mode) of **1** (80 μM) in H_2O:DMSO (10:1) at 37 ^oC in the presence of histidine (0.8 mM) after (A) 0 h and (B) 24 h incubation or cysteine (0.8 mM) after (C) 0 h and (D) 24 h incubation or glucose (0.8 mM) after (E) 0 h and (F) 24 h incubation.

Figure S23. ESI mass spectrum (positive mode) of **1** (80 μM) in H_2O:DMSO (10:1) at 37 ^oC in the presence of the cytoplasmic extract of HMLER-shEcad cells (0.5 million cells) after (A) 0 h and (B) 24 h incubation.

Figure S24. (A) Immunoblotting analysis of proteins related to the DNA damage response pathway. Protein expression in HMLER-shEcad cells following treatment with **1** (0.2-0.4 μM for 24 h). (C) Immunoblotting analysis of proteins related to caspase-dependent apoptosis. Protein expression in HMLER-shEcad cells following treatment with **1** (0.1-0.4 μM for 72 h).

Figure S25. FITC Annexin V-propidium iodide binding assay plots of (A) untreated HMLER-shEcad cells, (B) HMLER-shEcad cells treated with **1** (2 × IC_{50} value for 72 h), and (C) HMLER-shEcad cells treated with cisplatin (25 μM for 72 h).

Figure S26. Representative histograms displaying the green fluorescence emitted by anti-COX-2 Alexa Fluor 488 nm antibody-stained HMLER-shEcad cells without (red) and with (blue) LPS (2.5 μM) for 24 h, followed by 48 h in media.

Figure S27. (A) Representative histograms displaying the green fluorescence emitted by anti-COX-2 Alexa Fluor 488 nm antibody-stained HMLER-shEcad cells treated with LPS (2.5 μM) for 24 h, followed by 48 h in media (red) or media containing **1** (IC_{50} value, blue) or **1** (2 × IC_{50} value, orange). (B) Representative histograms displaying the green fluorescence emitted by anti-COX-2 Alexa Fluor 488 nm antibody-stained HMLER-shEcad cells treated with LPS (2.5 μM) for 24 h, followed by 48 h in media (red) or media containing flufenamic acid (20 μM, blue) or flufenamic acid (40 μM, orange).

Figure S28. Representative dose-response curves for the treatment of LPS pre-treated (2.5 µM for 24 h) HMLER-shEcad cells with **1** after 72 h incubation. Error bars represent standard deviations.

Figure S29. Immunoblotting analysis of high mobility group box 1 (HMGB-1). Protein expression in HMLER-shEcad cells following treatment with (A) **1** (0.1-0.4 µM for 72 h) or (B) **1** (2-10 µM for 24 h).

Figure S30. Chemical structure of the previously reported cobalt(III)-cyclam complex bearing two naproxen moieties, **3**.

Figure S31. Normalised extracellular ATP released from HMLER-shEcad cells untreated and treated with **3** (IC_{50} value, 2 × IC_{50} value, and 4 × IC_{50} value for 24 h). Error bars represent standard deviations.

Figure S32. Representative histograms displaying the green fluorescence emitted by anti-CRT Alexa Fluor 488 nm antibody-stained HMLER-shEcad cells untreated (red), and treated with **3** (IC_{50} value for 24 h) (blue) or **3** (2 × IC_{50} value for 24 h) (orange) or **3** (4 × IC_{50} value for 24 h) (green).

Figure S33. Immunoblotting analysis of high mobility group box 1 (HMGB-1). Protein expression in HMLER-shEcad cells following treatment with **3** (2-10 µM for 24 h).

Figure S34. Representative two-dimensional scatter plots of CellTracker Green-stained HMLER-shEcad cells (A) untreated and (B) treated with cisplatin (150 µM for 24 h) and thapsigargin (7 µM for 24 h) and then co-cultured with CellTracker Orange-stained THP-1 macrophages for 2 h. The population of HMLER-shEcad cells phagocytosed by THP-1 macrophages is indicated.

References

Experimental Details

Materials and methods. All synthetic procedures were performed under normal atmospheric conditions. ^{1}H, $^{31}P\{^{1}H\}$ and $^{19}F\{^{1}H\}$ NMR were recorded at room temperature on a Bruker Avance 400 spectrometer (^{1}H 400.0 MHz, ^{31}P 162.0 MHz, ^{19}F 376.5 MHz) with chemical shifts (δ, ppm) reported relative to the solvent peaks of the deuterated solvent. Fourier transform infrared (FTIR) spectra were recorded with an IRAffinity-1S Shimadzu spectrophotometer. UV-Vis absorption spectra were recorded on a Cary 3500 UV-Vis spectrophotometer. ICP-MS were measured using a Thermo Scientific ICAP-Qc quadrupole ICP mass spectrometer. Elemental analysis of the compounds prepared was performed commercially by the University of Cambridge. [Co(cyclam)Cl_2]Cl was prepared using a reported protocol.[1] Flufenamic acid, silver nitrate, and sodium sulphate were purchased from Sigma-Aldrich and used without further purification. Solvents were purchased from Fisher and used without further purification.

Synthesis of [Co(cyclam)(flufenamic acid)$_2$]PF_6 (1). Flufenamic acid (150 mg, 0.53 mmol) was dissolved in methanol (45 mL) dried with Na_2SO_4. [Co(cyclam)Cl_2]Cl (78 mg, 0.22 mmol) in methanol (5 mL), and Ag_2O (165 mg, 0.71 mmol) was added to this solution and the mixture was stirred for 2 days. The resulting silver salts were removed by filtration and the volume of the filtrate was reduced to ~5 mL. Diethyl ether (100 mL) was added and the resulting precipitate was collected and washed with diethyl ether (20 mL). The solid was suspended in acetone (10 mL), filtered, and the filtrate was removed under vacuum. The resultant residue was dissolved in the minimum of DMSO then dispersed in a solution of $NaPF_6$ (250 mg, 1.5 mmol) in water (10 mL). The resulting precipitate was collected and washed with water (2 × 10 mL). The crude product was further purified by Al_2O_3 column chromatography (DCM:MeOH (95:5)) to yield pure **1** as a tawny solid (88.5 mg, 42%). ^{1}H NMR (400 MHz, DMSO) δ 9.93 (s, 2H), 8.41 (br s, 4H), 7.55-7.50 (m, 4H), 7.45 (d, 2H), 7.40 (br t, 2H), 7.37-7.27 (m, 4H), 7.24 (dd, 2H), 6.84 (td, 2H), 3.01-2.99 (m, 4H), 2.72-2.68 (m, 4H), 2.56-2.52 (m, 6H), 2.41-2.34 (m, 4H), 2.24-2.20 (m, 2H); ^{31}P NMR (162 MHz, DMSO) δ -144.20 (sept, PF_6); ^{19}F NMR (376.5 MHz, DMSO) δ -61.30 (s, CF_3), -70.15 (d, PF_6); ATR-FTIR (solid, cm^{-1}): 3257, 3064, 2866, 1580, 1513, 1463, 1353, 1329, 1279, 1157, 1116, 1068, 997, 929, 837, 806, 792, 748, 697, 666, 582, 557, 511, 466, 411; ESI-MS Calcd. for $C_{38}H_{42}CoF_6N_6O_4$ [M-PF_6]$^+$: 819.2504 a.m.u. Found [M-PF_6]$^+$: 819.2504 a.m.u.; Anal. Calcd. for $C_{38}H_{42}CoF_{12}N_6O_4P$: C, 47.31; H, 4.39; N, 8.71. Found: C, 47.40; H, 4.44; N, 8.75.

X-ray crystallography. Crystals were mounted in inert oil on glass fibres and transferred to a Bruker Apex 2000 CCD area detector diffractometer. Data was collected using graphite-monochromated Mo-Kα radiation (λ = 0.71073) at 150(2) K. Scan type ϖ. Absorption corrections based on multiple scans were applied using SADABS[2] or spherical harmonics implemented in SCALE3 ABSPACK scaling algorithm.[3] The structures were solved by direct methods and refined on F^2 using the program SHELXT-2016.[4] All non-hydrogen atoms were refined anisotropically. The CCDC deposition number 2287973 contains the supplementary crystallographic data. This data can be obtained free of charge via The Cambridge Crystallography Data Centre.

Measurement of water-octanol partition coefficient (LogP). The LogP value for **1** was determined using the shake-flask method and UV-Vis spectroscopy. The 1-octanol used in this experiment was pre-saturated with water. A DMSO solution of **1** (10 μL, 10 mM) was incubated with 1-octanol (495 μL) and H_2O (495 μL) in a 1.5 mL tube. The tube was shaken

at room temperature for 24 h. The two phases were separated by centrifugation and the content of **1** in the water and 1-octanol phases was determined by UV-Vis spectroscopy.

Cell culture. The human mammary epithelial cell lines, HMLER and HMLER-shEcad were kindly donated by Prof. R. A. Weinberg (Whitehead Institute, MIT). HMLER and HMLER-shEcad cells were maintained in Mammary Epithelial Cell Growth Medium (MEGM) with supplements and growth factors (BPE, hydrocortisone, hEGF, insulin, and gentamicin/amphotericin-B). The HEK 293 embryonic kidney cell line was acquired from American Type Culture Collection (ATCC, Manassas, VA, USA) and cultured in Dulbecco's Modified Eagle's Medium (DMEM) supplemented with 1% penicillin and 10% fetal bovine serum. The cells were grown at 310 K in a humidified atmosphere containing 5% CO2.

Cytotoxicity studies: MTT assay. Exponentially growing cells were seeded at a density of approximately 5×10^3 cells per well in 96-well flat-bottomed microplates and allowed to attach for 24 h prior to addition of compounds. Various concentrations of the test compounds (0.0004-100 μM) were added and incubated for 72 h at 37 °C (total volume 200 μl). Stock solutions of the compounds were prepared as 10 mM DMSO solutions and diluted using cell media. The final concentration of DMSO in each well was ≤1 %. After 72 h, 20 μl of MTT (4 mg ml^{-1} in PBS) was added to each well and the plates incubated for an additional 4 h at 37 °C. The media/MTT mixture was eliminated and DMSO (100 μl per well) was added to dissolve the formazan precipitates. The optical density was measured at 550 nm using a 96-well multiscanner autoreader. Absorbance values were normalised to (DMSO-containing) control wells and plotted as concentration of compound versus % cell viability. IC_{50} values were interpolated from the resulting dose dependent curves. The reported IC_{50} values are the average of three independent experiments (n = 18).

Tumorsphere formation and viability assay. HMLER-shEcad cells (5×10^3) were plated in ultralow-attachment 96-well plates (Corning) and incubated in MEGM supplemented with B27 (Invitrogen), 20 ng mL^{-1} EGF and 4 μg mL^{-1} heparin (Sigma) for 5 days. Studies were also conducted in the presence of **1**, **2**, flufenamic acid, cisplatin, and salinomycin (0-133 μM). Mammospheres treated with **1**, **2**, flufenamic acid, cisplatin, and salinomycin (at their respective IC_{20} values, 5 days) were counted and imaged using an inverted microscope. The viability of the mammospheres was determined by addition of a resazurin-based reagent, TOX8 (Sigma). After incubation for 16 h, the fluorescence of the solutions was read at 590 nm (λ_{ex} = 560 nm). Viable mammospheres reduce the amount of the oxidised TOX8 form (blue) and concurrently increase the amount of the fluorescent TOX8 intermediate (red), indicating the degree of mammosphere cytotoxicity caused by the test compound. Fluorescence values were normalised to DMSO-containing controls and plotted as concentration of test compound versus % mammospheres viability. IC_{50} values were interpolated from the resulting dose dependent curves. The reported IC_{50} values are the average of two independent experiments, each consisting of two replicates per concentration level (overall n = 4).

Cellular uptake. To measure the cellular uptake of **1** about 1 million HMLER-shEcad cells were treated with **1** (0.5 μM) at 37 °C for 24 h. After incubation, the media was removed, the cells were washed with PBS (2 mL × 3) and harvested. The number of cells was counted at this stage, using a haemocytometer. This mitigates any cell death induced by **1** at the administered concentration and experimental cell loss. Half of the cellular pellet was dissolved in 65% HNO_3 (250 μL) overnight. Half of the cellular pellet was used to determine the cobalt content in the cytoplasmic, nuclear, and membrane fractions. The Thermo

Scientific NE-PER Nuclear and Cytoplasmic Extraction Kit was used to extract and separate the cytoplasmic, nuclear, and membrane fractions. The fractions were dissolved in 65% HNO_3 (250 µL final volume) overnight. All samples were diluted 17-fold with water and analysed using inductively coupled plasma mass spectrometry (ICP-MS, Thermo Scientific ICAP-Qc quadrupole ICP mass spectrometer). Cobalt levels are expressed as mass of Co (ng) per million cells. Results are presented as the mean of four determinations for each data point.

Immunoblotting analysis. HMLER-shEcad cells (5×10^6) were incubated with **1** (0.2-0.8 µM for 24 h or 0.1-0.4 µM for 72 h) at 37 °C. HMLER-shEcad cells were harvested and isolated as pellets. SDS-PAGE loading buffer (64 mM Tris-HCl (pH 6.8), 9.6% glycerol, 2% SDS, 5% β-mercaptoethanol, 0.01% bromophenol blue) was added to the pellets, and this was incubated at 95 °C for 10 min. Cell lysates were resolved by 4-20 % sodium dodecylsulphate polyacylamide gel electrophoresis (SDS-PAGE; 200 V for 25 min) followed by electro transfer to polyvinylidene difluoride membrane, PVDF (350 mA for 1 h). Membranes were blocked in 5% (w/v) non-fat milk in PBST (PBS/0.1% Tween 20) and incubated with the appropriate primary antibodies (Cell Signalling Technology). After incubation with horseradish peroxidase-conjugated secondary antibodies (Cell Signalling Technology), immune complexes were detected with the ECL detection reagent (BioRad) and analysed using a chemiluminescence imager (Bio-Rad ChemiDoc Imaging System).

Annexin V-propidium iodide assay. HMLER-shEcad cells were incubated with and without **1** ($2 \times IC_{50}$ value or $4 \times IC_{50}$ value for 72 h) and cisplatin (25 µM for 72 h) at 37 °C. Cells were harvested from adherent cultures by trypsinisation. The FITC Annexin V/Dead Cell Apoptosis Kit was used. The manufacture's (Thermo Fisher Scientific) protocol was followed to carry out this experiment. Briefly, untreated and treated cells (1×10^6) were suspended in 1× Annexin binding buffer (100 µL) (10 mM HEPES, 140 mM NaCl, 2.5 mM $CaCl_2$, pH 7.4), then 5 µL FITC Annexin V and 1 µL PI (100 µg/ mL) were added to each sample and incubated at room temperature for 15 min. After which more 1× Annexin binding buffer (400 µL) was added while gently mixing. The cells were analysed using a FACSCanto II flow cytometer (BD Biosciences) (10,000 events per sample were acquired) at the University of Leicester FACS Facility. The FL1 channel was used to assess Annexin V binding and the FL2 channel was used to assess PI uptake. Cell populations were analysed using the FlowJo software (Tree Star).

COX-2 expression assay. HMLER-shEcad cells were seeded in 6-well plates (at a density of 5×10^5 cells/ mL) and the cells were allowed to attach overnight. The cells were treated with lipopolysaccharide (LPS) (2.5 µM for 24 h), and then treated with **1** (IC_{50} value or $2 \times IC_{50}$ value) or flufenamic acid (20 or 40 µM) and incubated for a further 48 h. The cells were then harvested by trypsinisation, fixed with 4% paraformaldehyde (at 37 °C for 10 min), permeabilised with ice-cold methanol (for 30 min), and suspended in PBS (200 µL). The Alexa Fluor® 488 nm labelled anti-COX-2 antibody (2 µL) was then added to the cell suspension and incubated in the dark for 1 h. The cells were then washed with PBS (1 mL) and analysed using a FACSCanto II flow cytometer (BD Biosciences) (10,000 events per sample were acquired) at the University of Leicester FACS Facility. The FL1 channel was used to assess COX-2 expression. Cell populations were analysed using the FlowJo software (Tree Star).

CRT cell surface exposure. Flow cytometry was used to analyse cell surface CRT exposure. HMLER-shEcad cells were seeded into a 6-well plate (at a density of 5×10^5 cells/ mL) and

the cells were incubated at 37 °C overnight. The cells were treated with **1** (IC_{50} value, 2 × IC_{50} value or 4 × IC_{50} value), **3** (IC_{50} value, 2 × IC_{50} value or 4 × IC_{50} value) or co-treated with cisplatin (150 μM) with thapsigargin (7 μM) for 24 h at 37 °C. The cells were then harvested by trypsinization and collected by centrifugation. The pellet was suspended in PBS (500 μL), and after the addition of the Alexa Fluor® 488 nm labelled anti-CRT antibody (2 μl), the cells were incubated in the dark for 30 minutes. The cells were then washed with PBS (1 mL) and analysed using a FACSCanto II flow cytometer (BD Biosciences) (10,000 events per sample were acquired) at the University of Leicester FACS Facility. The FL1 channel was used to assess CRT cell surface exposure. Cell populations were analysed using the FlowJo software (Tree Star).

ATP assay. HMLER-shEcad cells (5 × 10^3 cells /well) were seeded in a 96-well plate and incubated overnight. The cells were then treated with **1** (IC_{50} value, 2 × IC_{50} value or 4 × IC_{50} value), **3** (IC_{50} value, 2 × IC_{50} value, and 4 × IC_{50} value) or cisplatin (50 μM, positive control) for 24 h at 37 °C. The media was carefully extracted and transferred into a white-walled opaque 96-well plate, and a luciferin-based ENLITEN ATP Assay Kit (Promega) was used to measure the relative amount of ATP released into the supernatant.

HMGB-1 release. HMLER-shEcad cells (1 × 10^6 cells) were incubated with **1** (0.1-0.4 μM) for 72 h, **1** (2-10 μM) for 24 h or **3** (2-10 μM) for 24 h at 37 °C. Cells were collected in full and added to SDS-PAGE loading buffer (64 mM Tris-HCl (pH 6.8), 9.6% glycerol, 2% SDS, 5% β-mercaptoethanol, 0.01% Bromophenol Blue) and incubated at 95 °C for 10 min. The HMGB-1 content was probed by immunoblotting analysis as described above. The anti-HMGB-1 antibody (Cell Signalling Technology) was used in this experiment.

Phagocytosis assay. HMLER-shEcad cells were seeded into a 6-well plate (at a density of 5 × 10^5 cells/ mL) and the cells were incubated at 37 °C overnight. The cells were stained with CellTracker Green (30 min) and washed with MEGM media. The cells were then treated with **1** (5 μM), **2** (20 μM), flufenamic acid (20 μM) or cisplatin (150 μM) with thapsigargin (7 μM) for 24 h at 37 °C. Then macrophages, obtained by differentiating THP-1 cells with phorbol 12-myristate 13-acetate (100 nM for 72 h) and pre-stained with CellTracker Orange for 30 min and washed with RPMI 1640 media, were added to the HMLER-shEcad cells (at a density of 1 × 10^5 cells/ mL). After 2 h, phagocytosis was assessed by flow cytometry, using a FACSCanto II flow cytometer (BD Biosciences) (10,000 events per sample were acquired) at the University of Leicester FACS Facility. The FL1 channel was used to assess the CellTracker Green-stained HMLER-shEcad cell population and the FL4 channel was used to assess the CellTracker Orange-stained THP-1 macrophage population. Cell populations were analysed using the FlowJo software (Tree Star).

Mice. Female, 5-week-old BALB/c mice were purchased from Charles River Laboratories (Wilmington, MA). All mice were quarantined for one week before use and kept in micro-isolator cages (four mice per cage) in a temperature- and humidity-controlled environment as per the Division of Laboratory Animal Research (DLAR) of University of Kentucky. All mice were maintained in a pathogen-free environment under the care of DLAR of University of Kentucky. Our study was performed in compliance with the NIH guidelines (NIH Publication No. 85-23 Rev. 1985) for the care and use of laboratory animals and all experimental procedures were monitored and approved by the Institutional Animal Care and Use Committee (IACUC #2019-3183; PI - S.G.A.) of the University of Kentucky (USA).

***In vivo* studies with 1**. Ten female BALB/c mice (5 weeks) were received from Charles River Laboratories (Wilmington, MA). They had an acclimation period of one week before being implanted with 4T1 cells (1 million) subcutaneously on their right flank. Three days post-implantation, the mice were administered with 10 mg/kg of **1** intraperitoneally (IP), 0.1 mL/mouse formulated as 1% DMSO, 37.5% Kolliphor, 37.5% ethanol, and 24% DI water. The control group was injected with a PBS solution containing 1% DMSO and 1% Kolliphor. The injection of **1**, and tumour-size and body-weight measurements were performed three days a week, and the mice were euthanised after 14 days (n = 5 for **1** and n = 5 for vehicle control).

Hematoxylin and eosin staining. The mice used in the *in vivo* comparative experiment of **1** and the vehicle control were sacrificed at day 17 post tumour cells (4T1) injection (day 14 after the first **1** or vehicle injections). The tissues (tumour, lungs, heart, liver, spleen, and kidney) were fixed in freshly prepared paraformaldehyde (4% in PBS) for 24 h and processed for paraffin sectioning. Tissue sections of 5 µm were stained with hematoxylin and eosin and used for histological examination. A total of 3 sections per tissue (spanning the full depth of the tissue) were examined and photographed using a Nikon Eclipse 55i microscope.

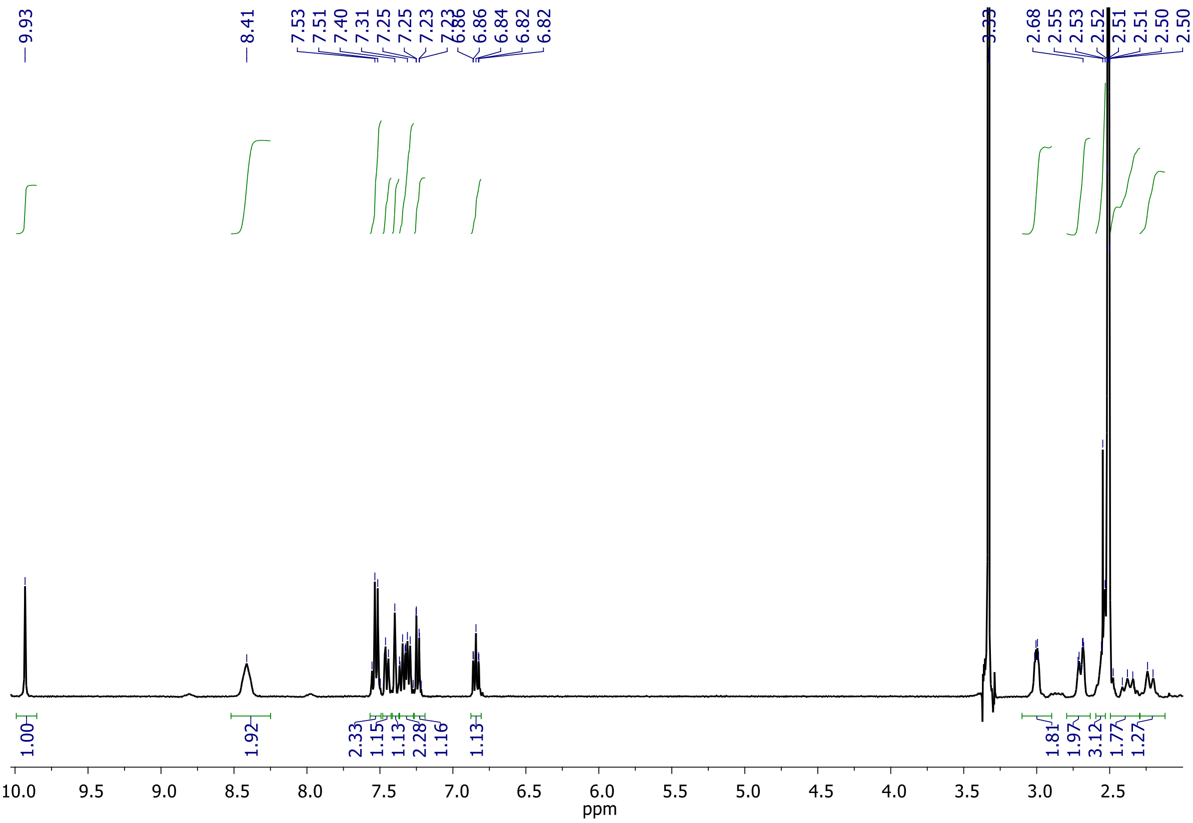

Figure S1. ^{1}H NMR spectrum of **1** in DMSO-d_6.

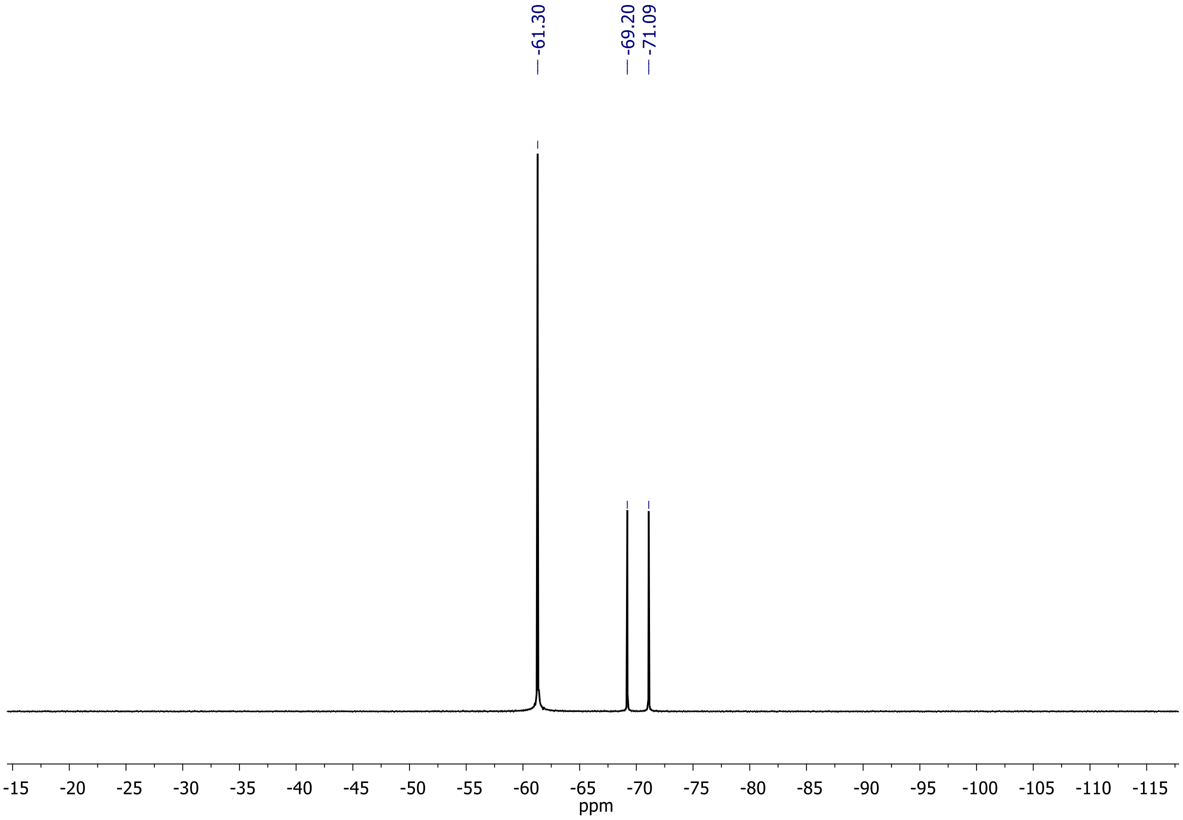

Figure S2. $^{19}F\{^{1}H\}$ NMR spectrum of **1** in DMSO-d_6.

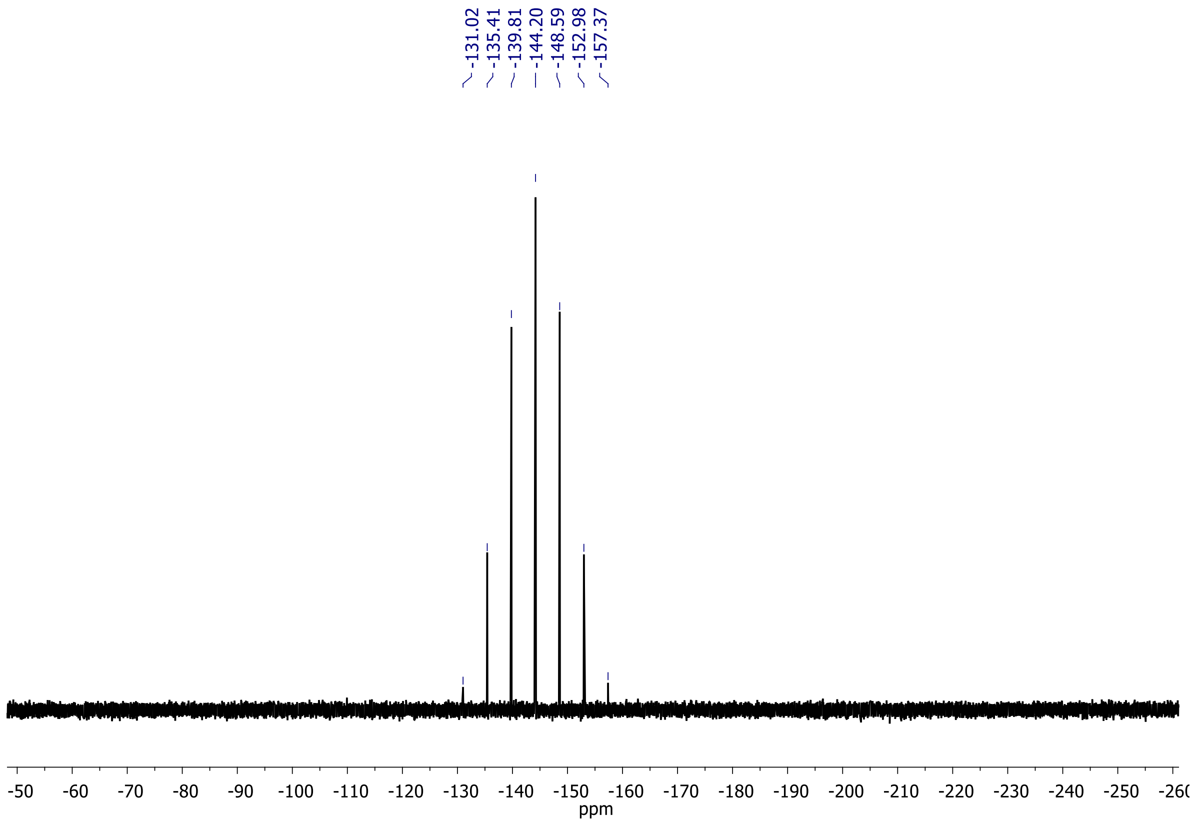

Figure S3. $^{31}P\{^{1}H\}$ NMR spectrum of **1** in DMSO-d_6.

.

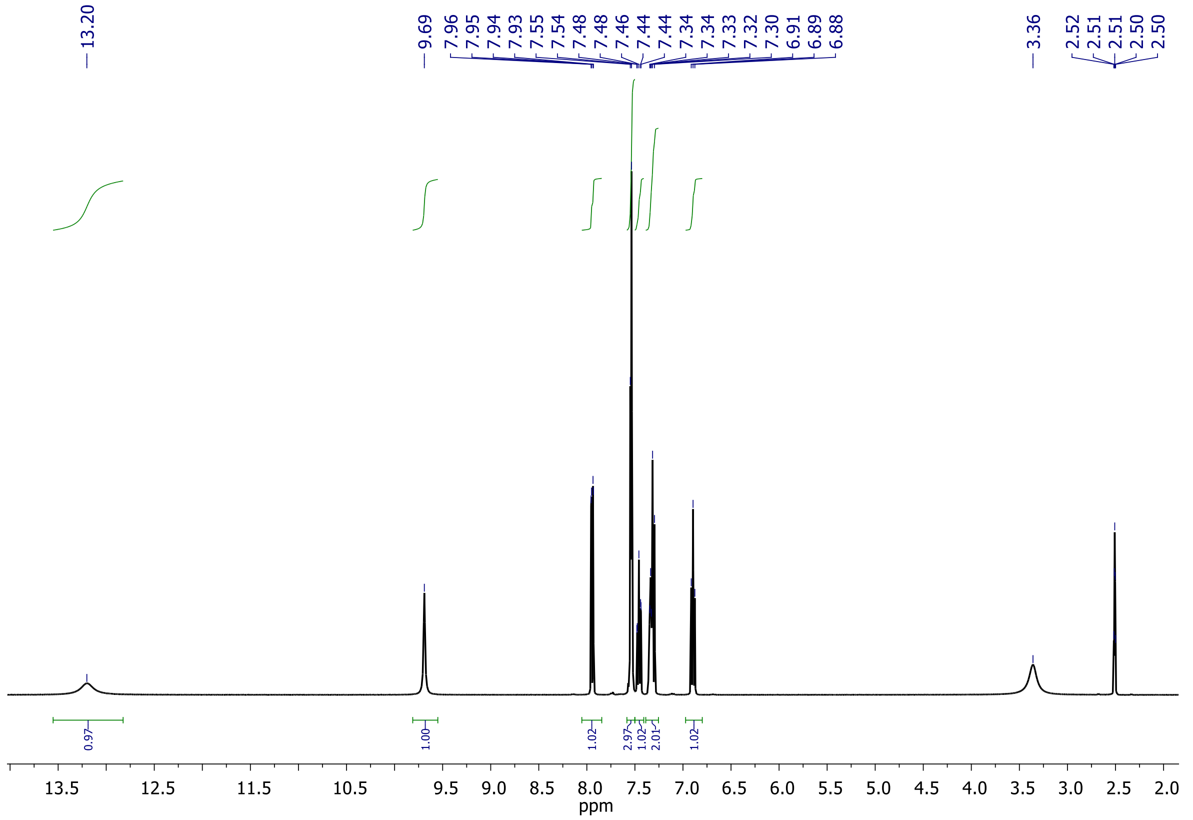

Figure S4. ^{1}H NMR spectrum of flufenamic acid in DMSO-d_6.

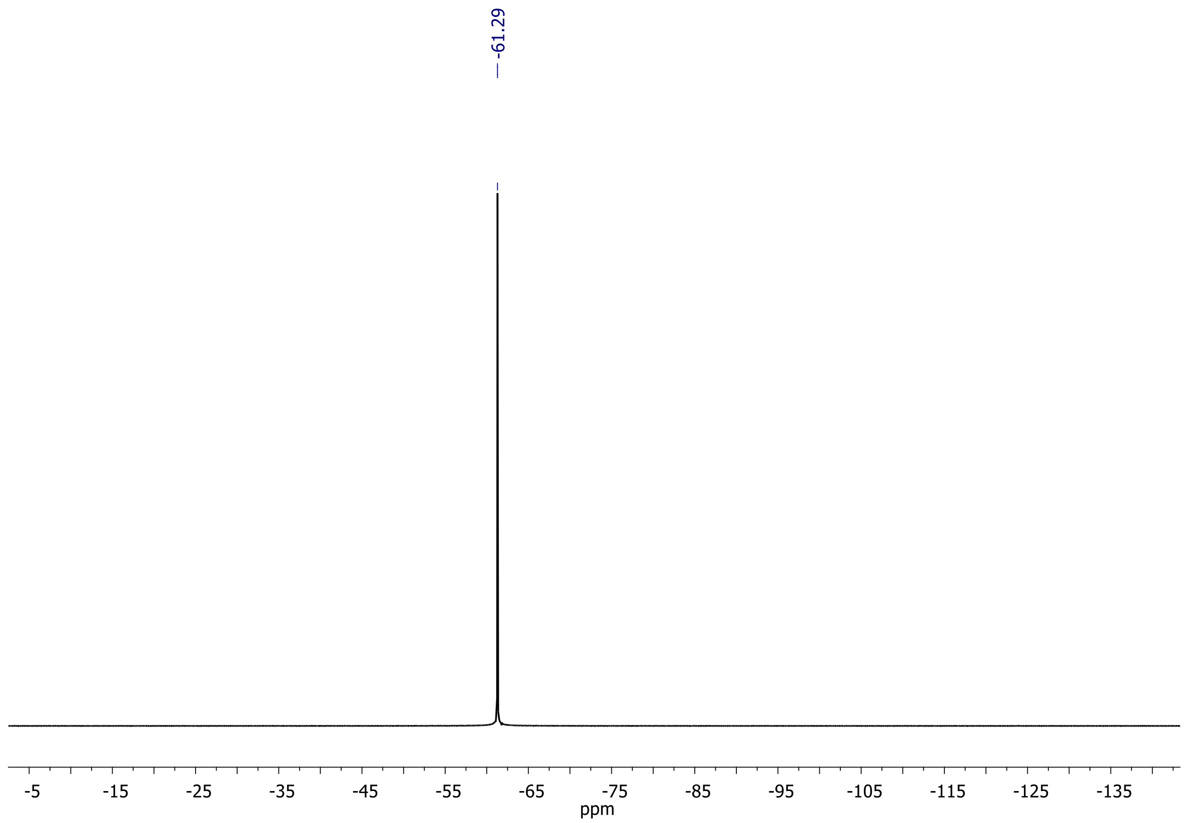

Figure S5. $^{19}F\{^{1}H\}$ NMR spectrum of flufenamic acid in DMSO-d_6.

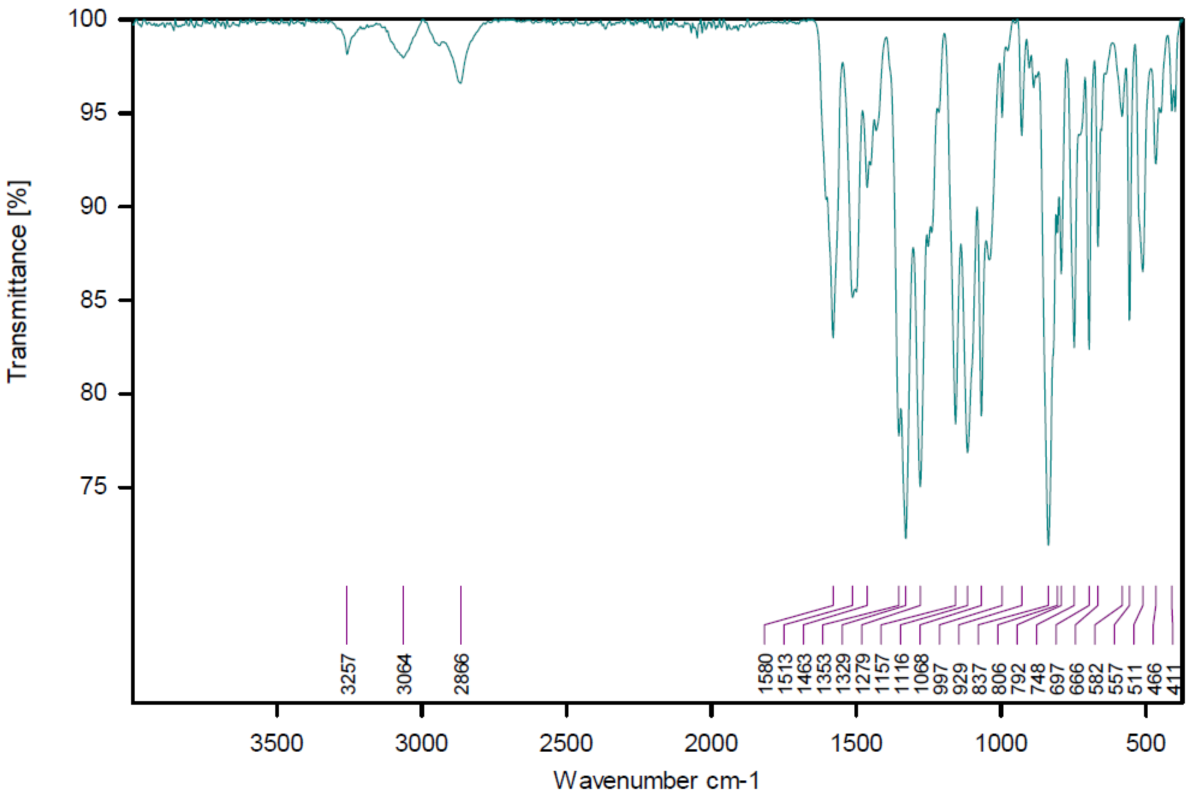

Figure S6. ATR-FTIR spectra of **1** in the solid form.

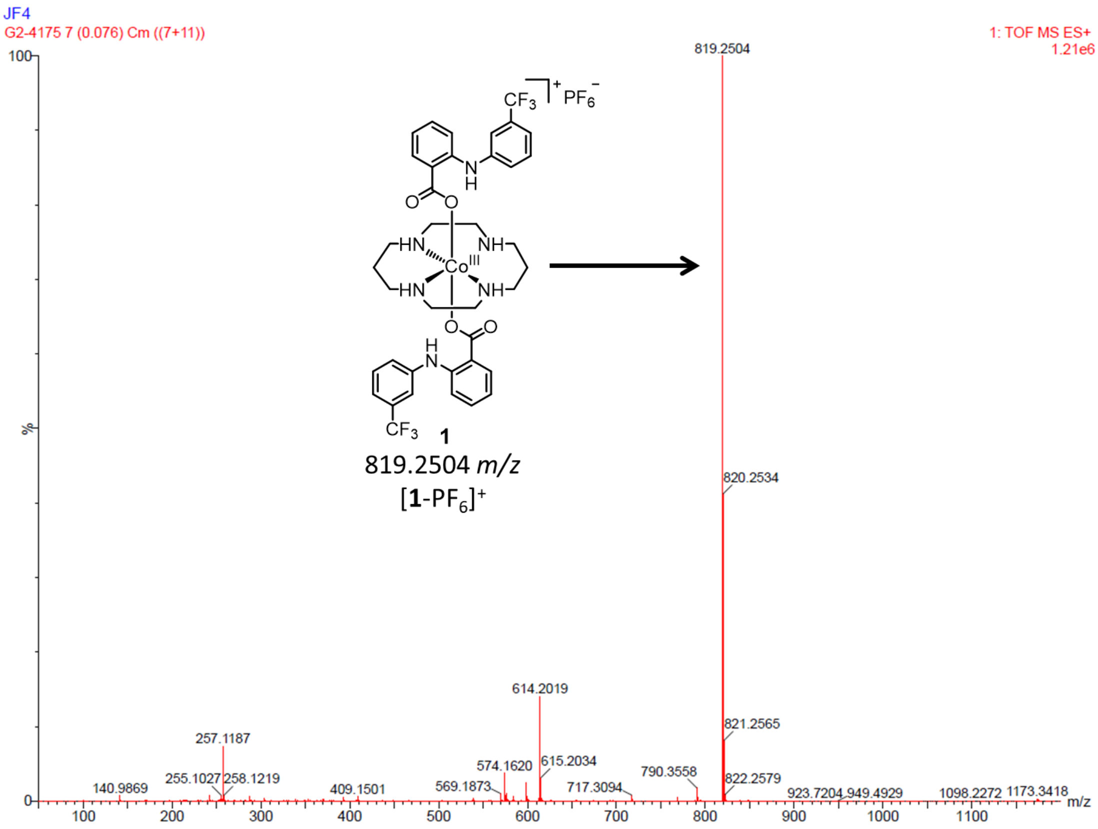

Figure S7. High resolution ESI mass spectrum (positive mode) of **1**.

Table S1. Crystallographic data for cobalt(III) complex **1**.

**Metal complex**	**1**
CCDC No.	2287973
formula	$C_{80}H_{94}Cl_2Co_2F_{12}N_{12}O_9$
Fw	1784.43
Crystal system	monoclinic
Space group	$P2_1$
a, Å	11.7728(2)
b, Å	22.3687(4)
c, Å	16.5602(3)
α, deg.	90
β, deg.	101.3310(10)
γ, deg.	90
V, Å^3	4276.00(13)
Z	2
D_{calcd}, Mg/m^3	1.386
2θ / deg.	5.442 to 136.462
Reflections collected	57418
Independent reflections	14546
Goodness-of-fit on F^2	1.036
R_1, wR_2 [$I \geq 2\sigma(I)$]	0.0569, 0.1631
R_1, wR_2 [all data]	0.0760, 0.1778

Table S2. Selected bond lengths (Å) and angles (°) for cobalt(III) complex **1**.

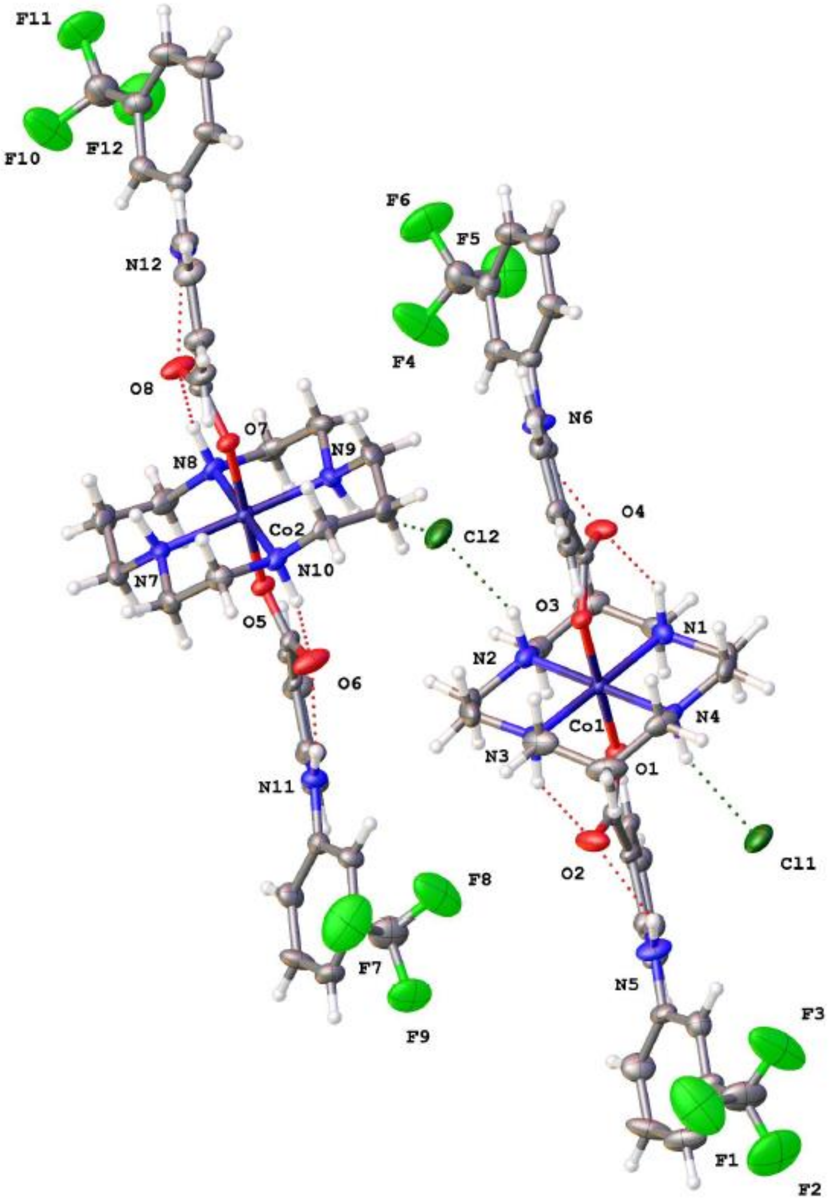

Co(1)-O(1)	1.923(5)	Co(2)-N(9)	1.966(5)
Co(1)-O(3)	1.931(5)	Co(2)-N(10)	1.977(6)
Co(1)-N(1)	1.971(6)	Co(2)-O(5)	1.924(5)
Co(1)-N(2)	1.961(6)	Co(2)-O(7)	1.924(5)
Co(1)-N(3)	1.974(6)	Co(2)-N(7)	1.961(5)
Co(1)-N(4)	1.962(5)	Co(2)-N(8)	1.969(5)
O(1)-Co(1)-O(3)	179.1(3)	O(5)-Co(2)-N(9)	89.5(2)

O(1)-Co(1)-N(1)	85.4(2)	O(5)-Co(2)-N(10)	94.9(2)
O(1)-Co(1)-N(2)	89.1(2)	O(7)-Co(2)-N(7)	89.4(2)
O(1)-Co(1)-N(3)	95.2(2)	O(7)-Co(2)-N(8)	94.8(2)
O(1)-Co(1)-N(4)	90.5(2)	O(7)-Co(2)-N(9)	90.1(2)
O(3)-Co(1)-N(1)	94.8(2)	O(7)-Co(2)-N(10)	84.7(2)
O(3)-Co(1)-N(2)	91.8(2)	N(7)-Co(2)-N(8)	94.3(2)
O(3)-Co(1)-N(3)	84.6(2)	N(7)-Co(2)-N(9)	179.5(3)
O(3)-Co(1)-N(4)	88.6(2)	N(7)-Co(2)-N(10)	85.8(2)
N(1)-Co(1)-N(3)	179.3(3)	N(9)-Co(2)-N(8)	85.9(2)
N(2)-Co(1)-N(1)	93.1(2)	N(8)-Co(2)-N(10)	179.6(3)
N(2)-Co(1)-N(3)	86.7(3)	N(9)-Co(2)-N(10)	94.0(2)
N(2)-Co(1)-N(4)	179.4(3)	O(5)-Co(2)-O(7)	179.4(3)
N(4)-Co(1)-N(1)	86.5(3)	O(5)-Co(2)-N(7)	91.0(2)
N(4)-Co(1)-N(3)	93.8(3)	O(5)-Co(2)-N(8)	85.5(2)

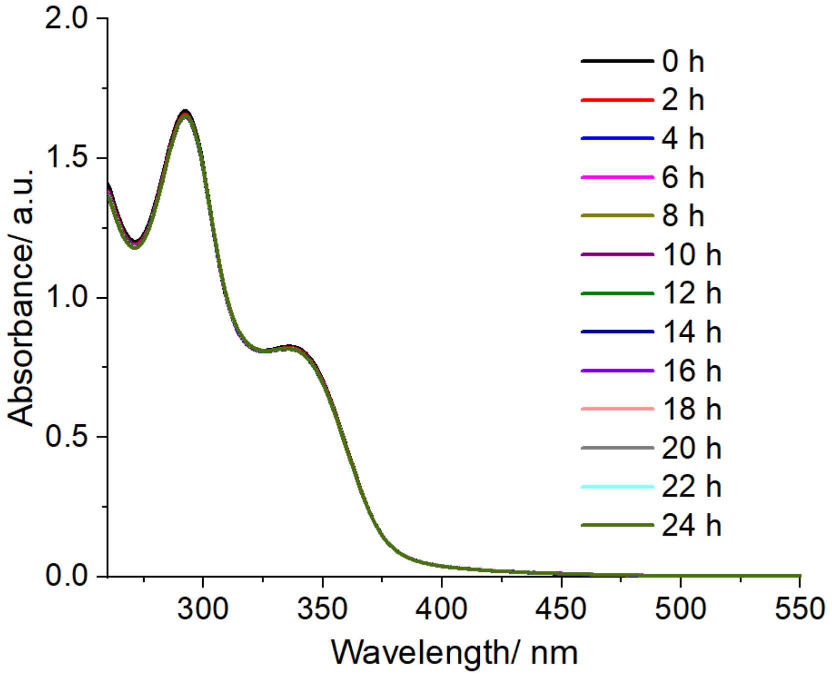

Figure S8. UV-Vis spectrum of **1** (50 µM) in DMSO over the course of 24 h at 37 °C.

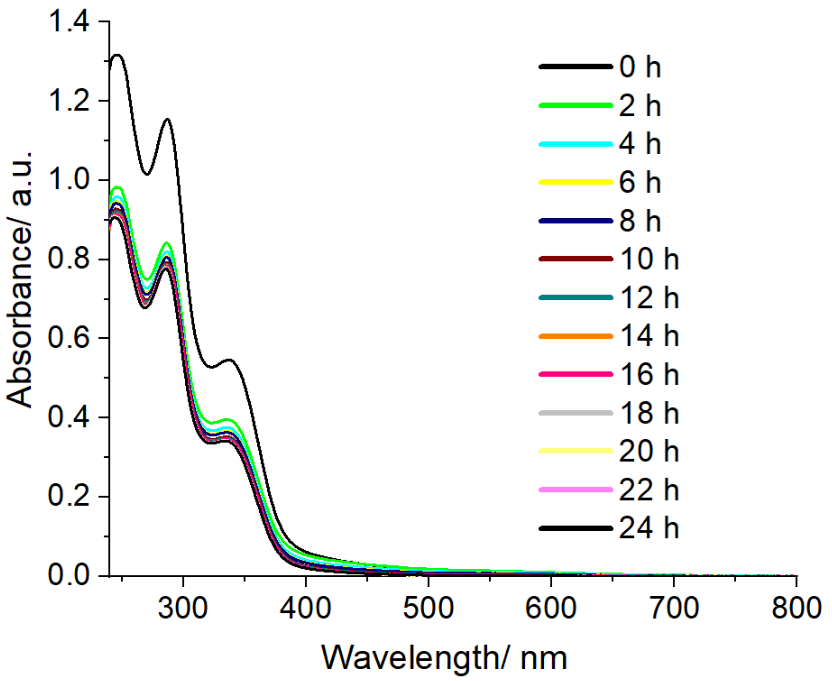

Figure S9. UV-Vis spectrum of **1** (50 µM) in H_2O:DMSO (200:1) over the course of 24 h at 37 °C.

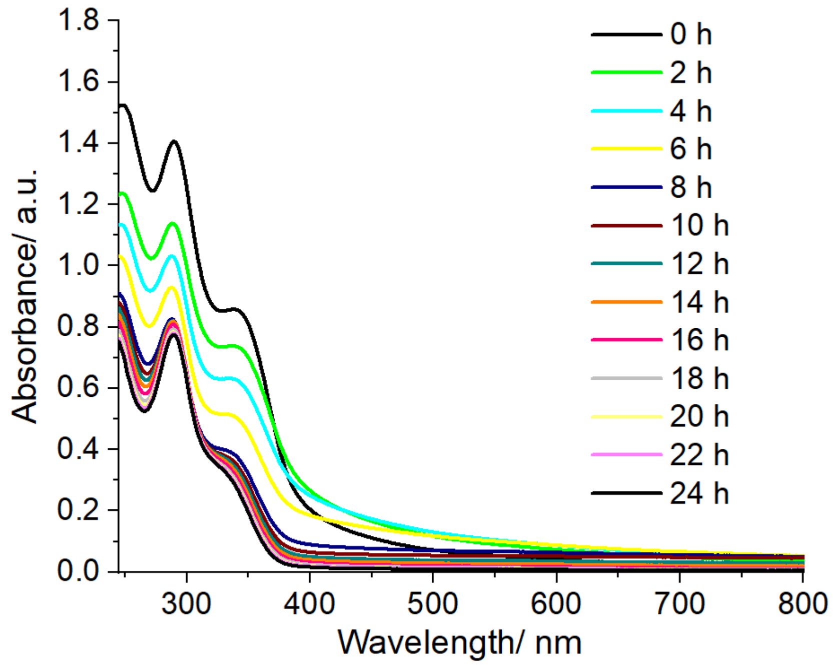

Figure S10. UV-Vis spectrum of **1** (50 μM) in PBS:DMSO (200:1) over the course of 24 h at 37 ^{o}C.

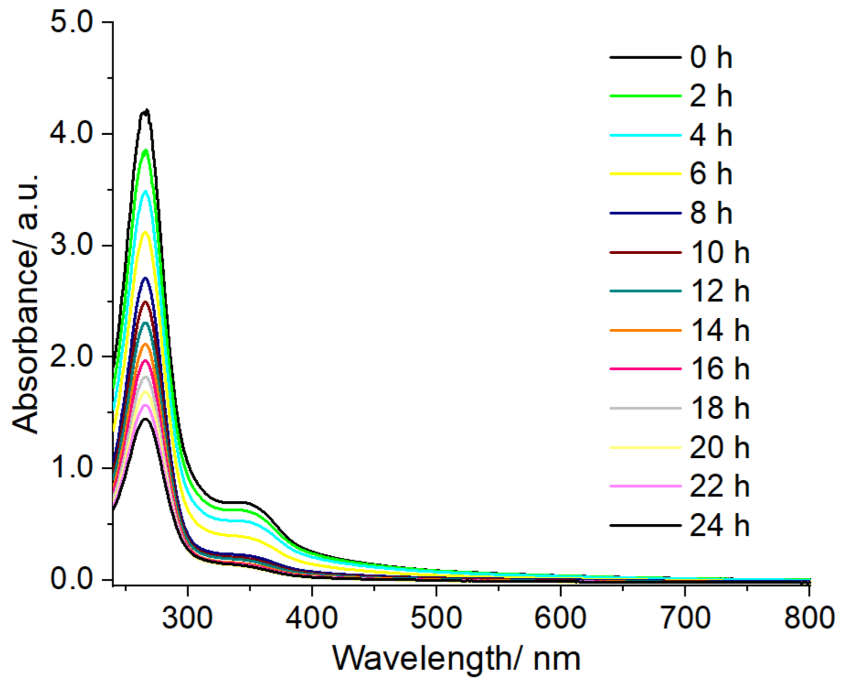

Figure S11. UV-Vis spectrum of **1** (50 μM) in the presence of ascorbic acid (500 μM) in H_2O:DMSO (200:1) over the course of 24 h at 37 ^{o}C.

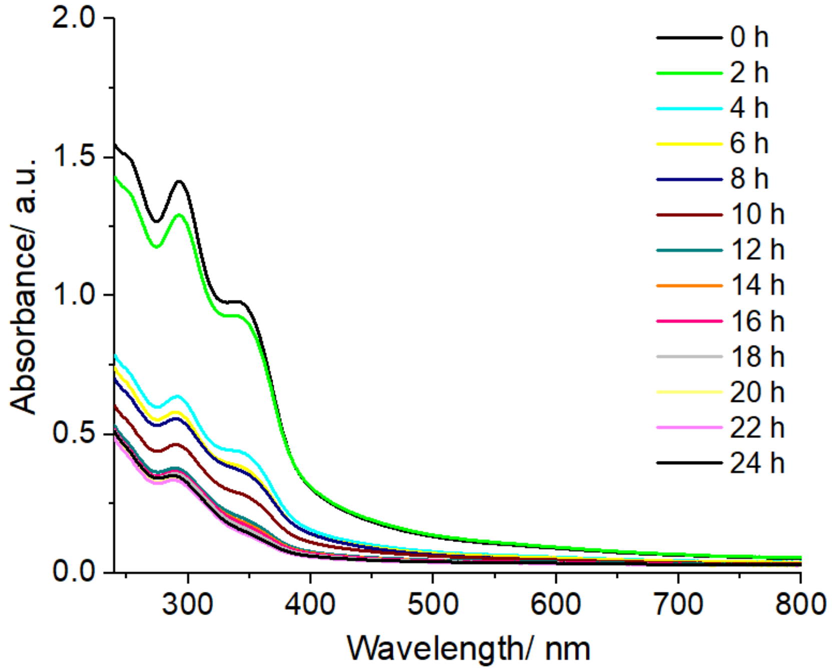

Figure S12. UV-Vis spectrum of **1** (50 μM) in the presence of glutathione (500 μM) in H_2O:DMSO (200:1) over the course of 24 h at 37 °C.

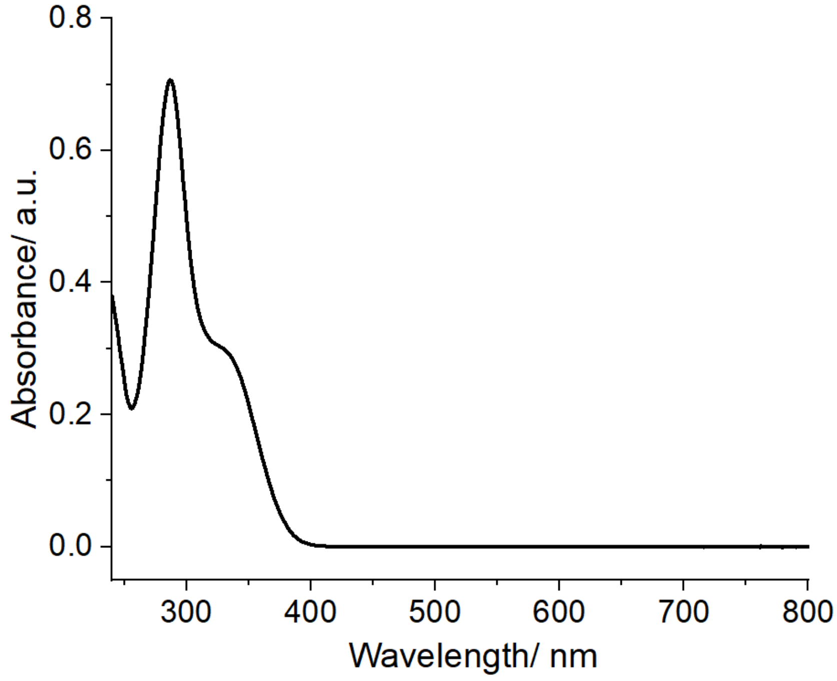

Figure S13. UV-Vis spectrum of flufenamic acid (50 μM) in H_2O:DMSO (200:1) at 37 °C.

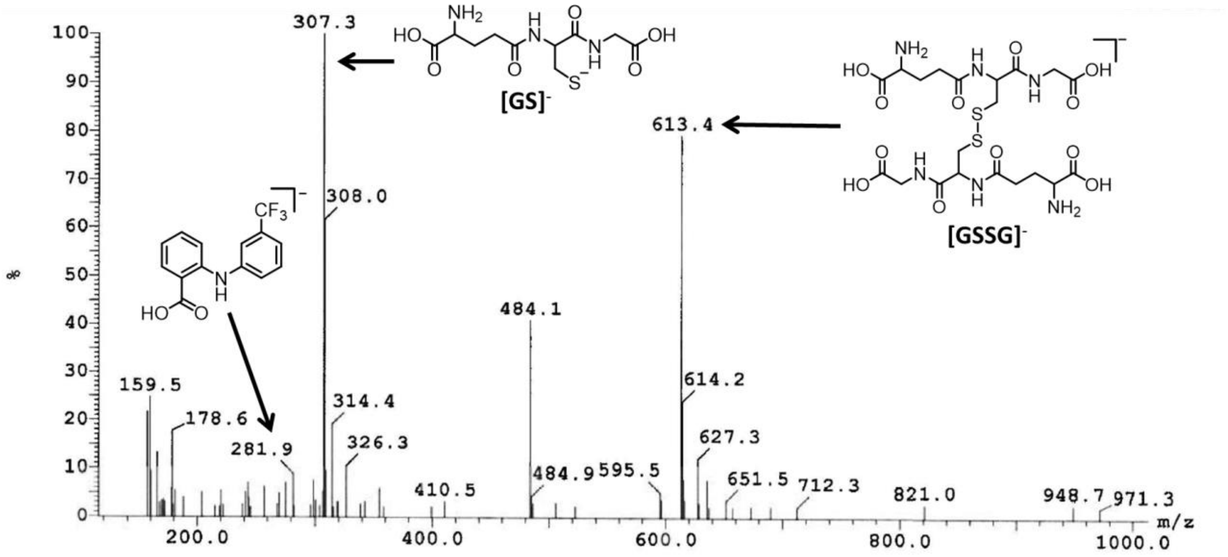

Figure S14. ESI mass spectrum (negative mode) of **1** (40 μM) in H_2O:DMSO (10:1), in the presence of glutathione (400 μM) after 24 h.

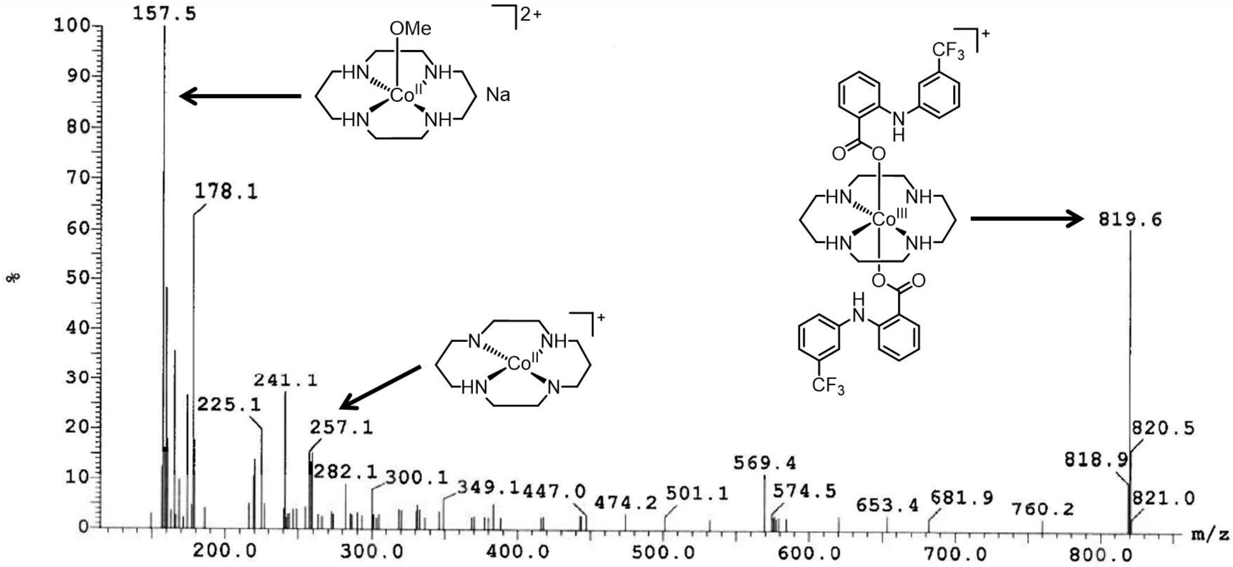

Figure S15. ESI mass spectrum (positive mode) of **1** (40 μM) in H_2O:DMSO (10:1), in the presence of glutathione (400 μM) after 24 h.

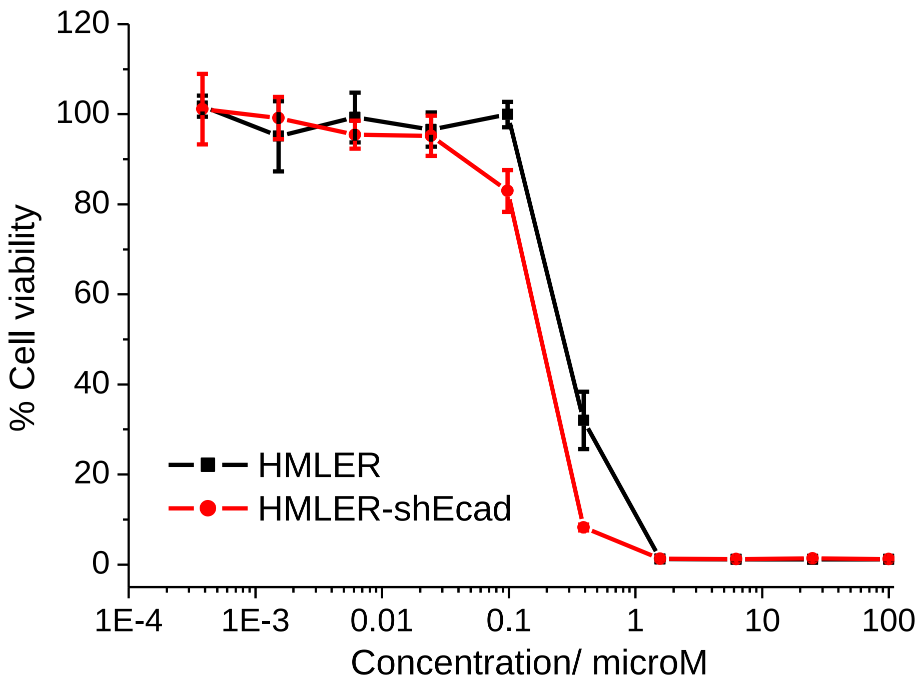

Figure S16. Representative dose-response curves for the treatment of HMLER and HMLER-shEcad cells with **1** after 72 h incubation. Error bars represent standard deviations.

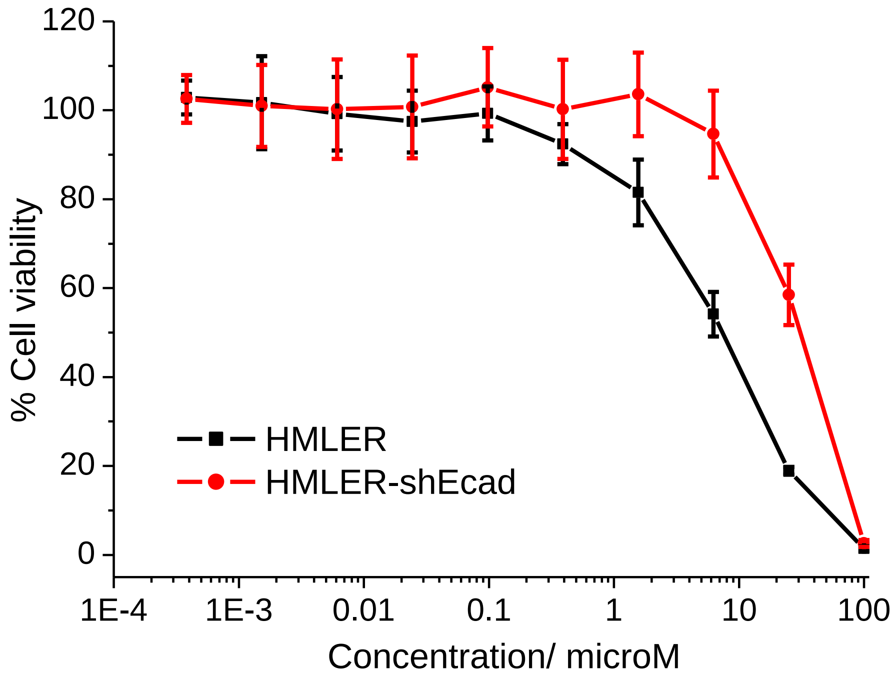

Figure S17. Representative dose-response curves for the treatment of HMLER and HMLER-shEcad cells with flufenamic acid after 72 h incubation. Error bars represent standard deviations.

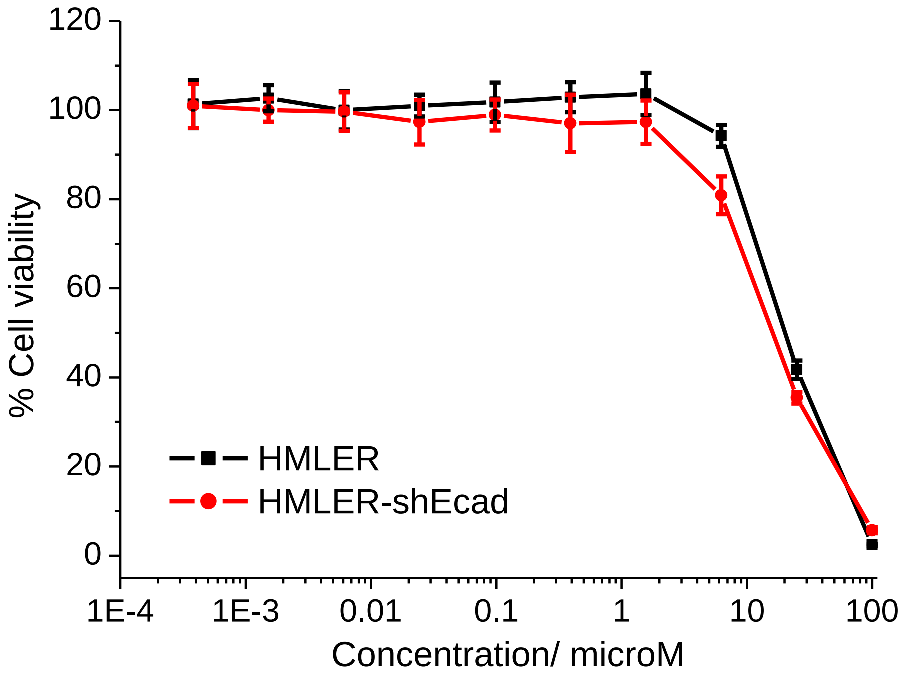

Figure S18. Representative dose-response curves for the treatment of HMLER and HMLER-shEcad cells with a mixture of **2** and flufenamic acid (1:2) after 72 h incubation. Error bars represent standard deviations.

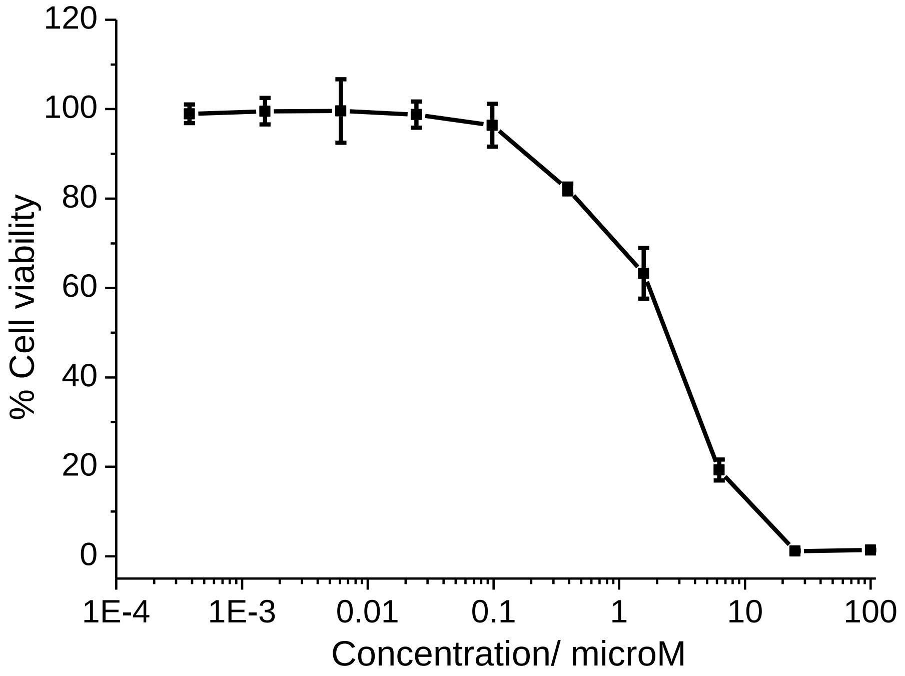

Figure S19. Representative dose-response curves for the treatment of HEK 293 cells with **1** after 72 h incubation. Error bars represent standard deviations.

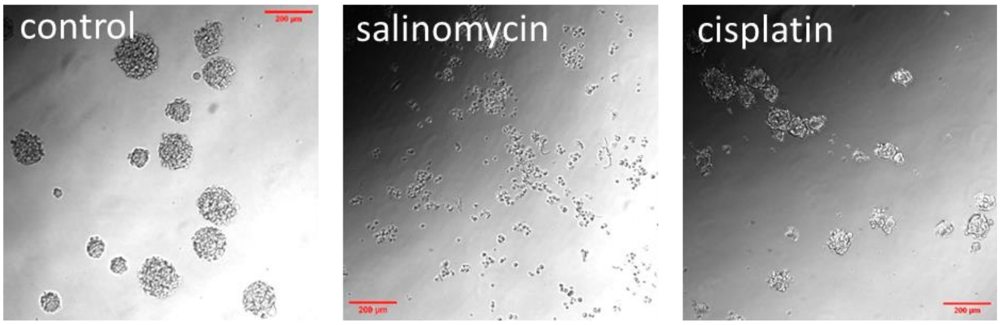

Figure S20. Representative bright-field images (× 10) of HMLER-shEcad mammospheres in the absence and presence of salinomycin or cisplatin at their IC_{20} values (5 days incubation).

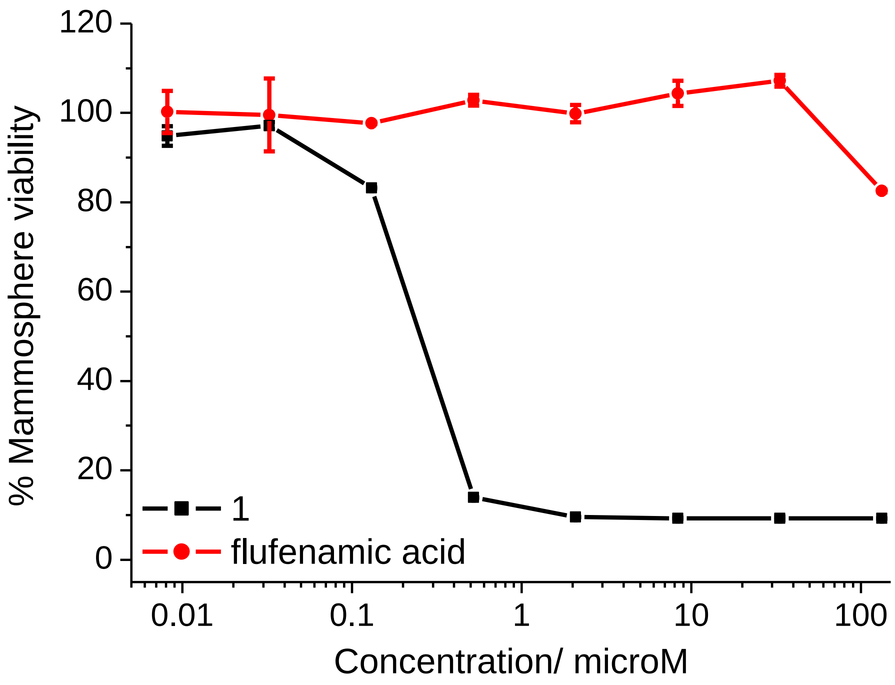

Figure S21. Representative dose-response curves for the treatment of HMLER-shEcad mammospheres with **1** or flufenamic acid after 5 days incubation. Error bars represent standard deviations.

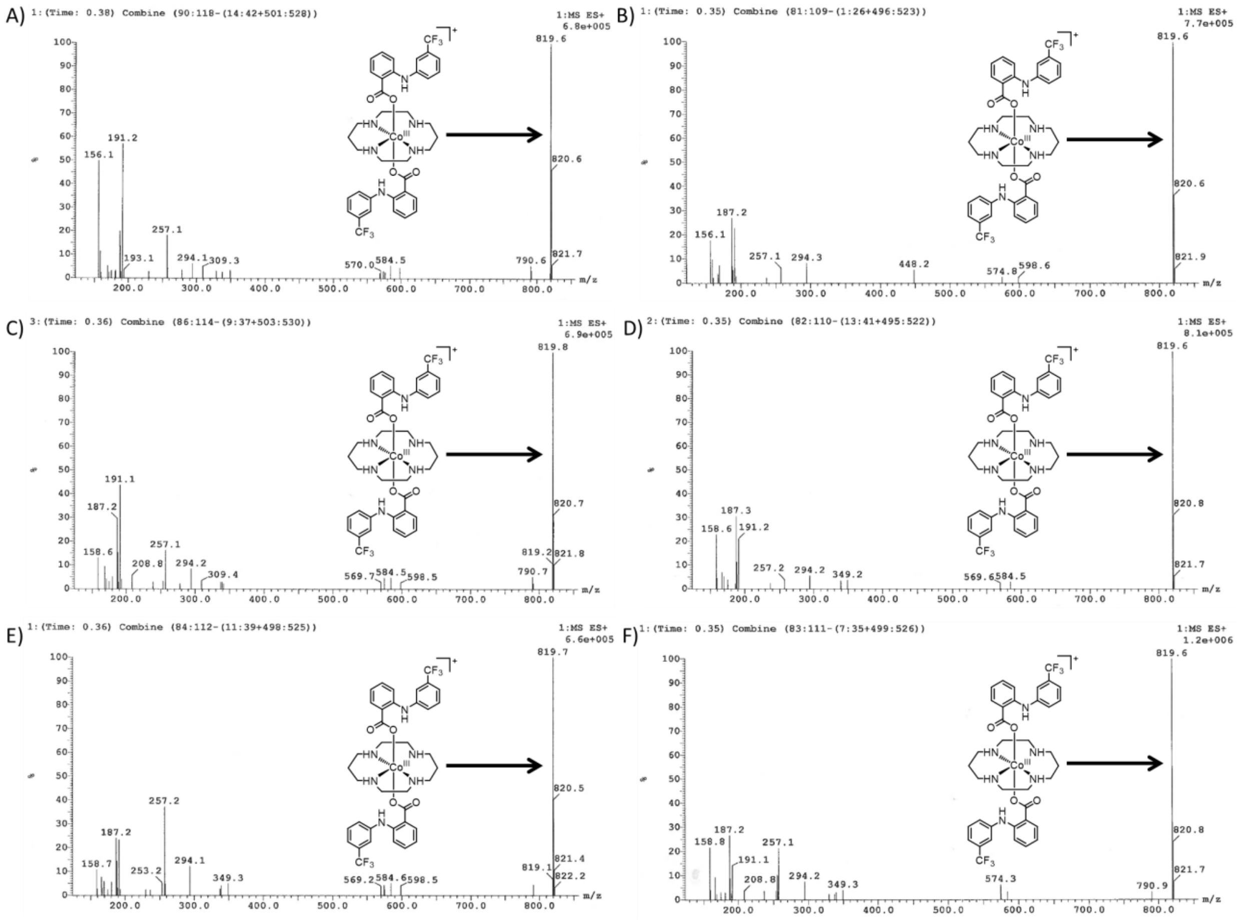

Figure S22. ESI mass spectrum (positive mode) of **1** (80 μM) in H_2O:DMSO (10:1) at 37 °C in the presence of histidine (0.8 mM) after (A) 0 h and (B) 24 h incubation or cysteine (0.8 mM) after (C) 0 h and (D) 24 h incubation or glucose (0.8 mM) after (E) 0 h and (F) 24 h incubation.

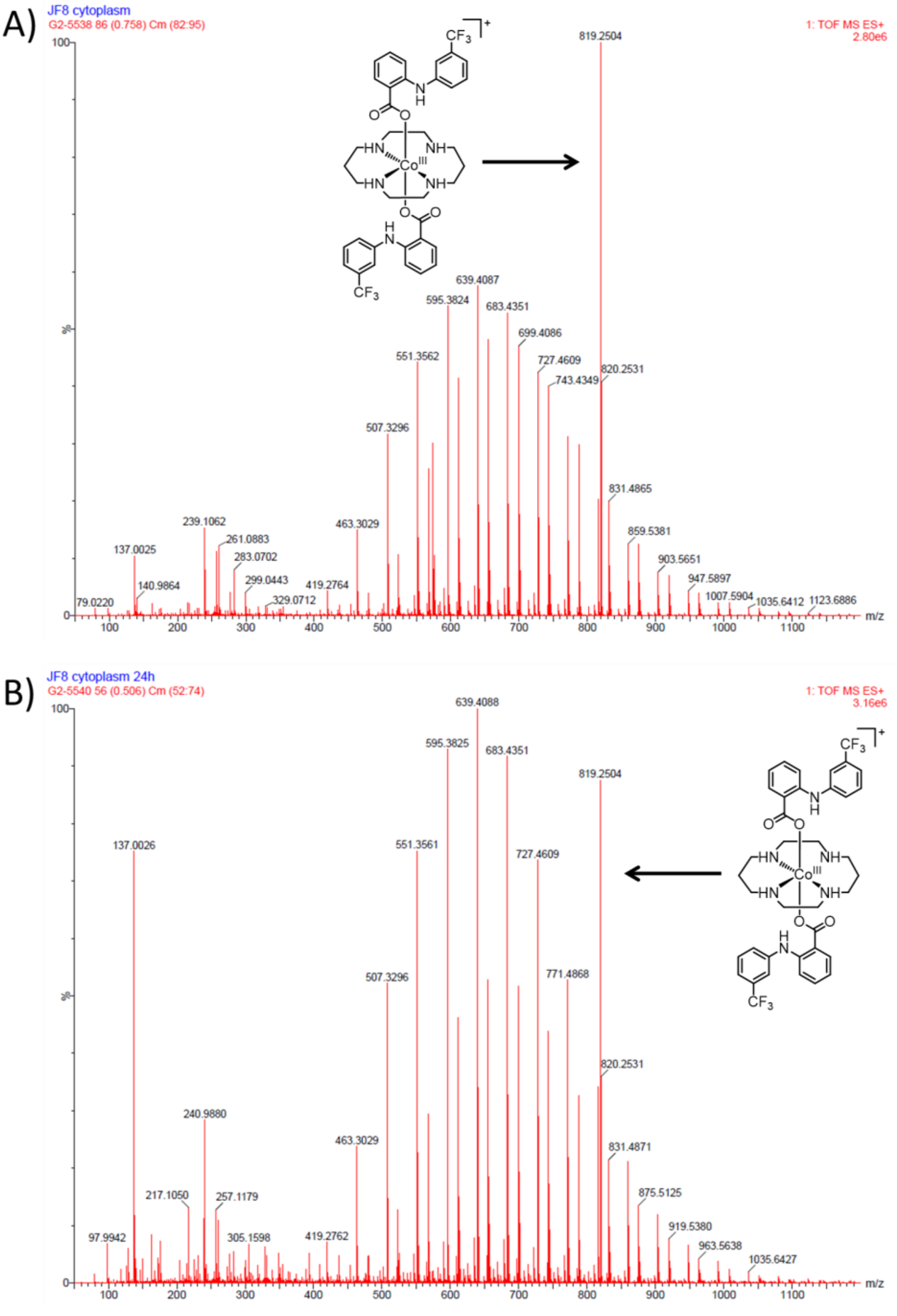

Figure S23. ESI mass spectrum (positive mode) of **1** (80 μM) in H_2O:DMSO (10:1) at 37 ^{o}C in the presence of the cytoplasmic extract of HMLER-shEcad cells (0.5 million cells) after (A) 0 h and (B) 24 h incubation.

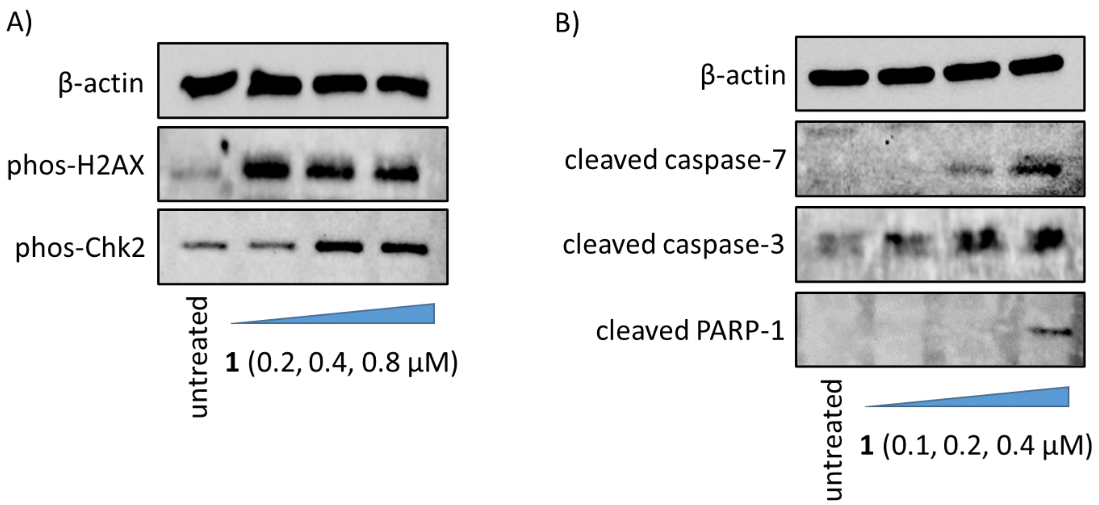

Figure S24. (A) Immunoblotting analysis of proteins related to the DNA damage response pathway. Protein expression in HMLER-shEcad cells following treatment with **1** (0.2-0.4 μM for 24 h). (C) Immunoblotting analysis of proteins related to caspase-dependent apoptosis. Protein expression in HMLER-shEcad cells following treatment with **1** (0.1-0.4 μM for 72 h).

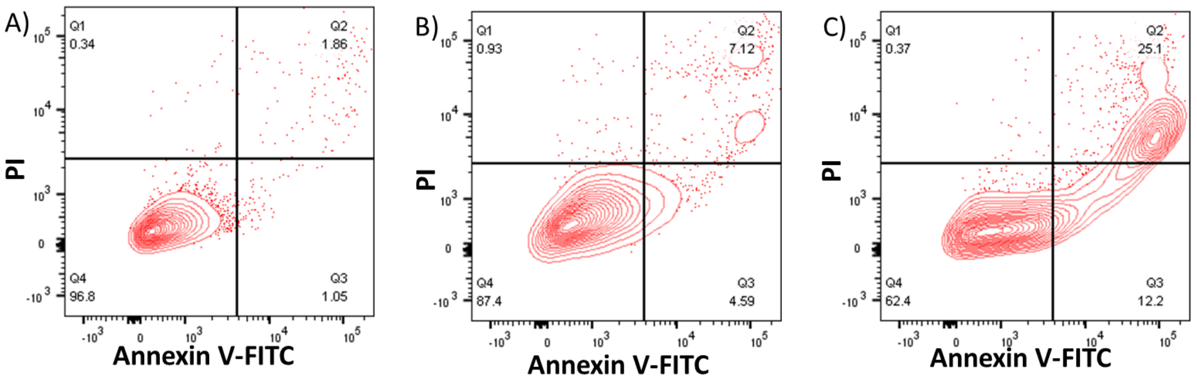

Figure S25. FITC Annexin V-propidium iodide binding assay plots of (A) untreated HMLER-shEcad cells, (B) HMLER-shEcad cells treated with **1** (2 × IC_{50} value for 72 h), and (C) HMLER-shEcad cells treated with cisplatin (25 μM for 72 h).

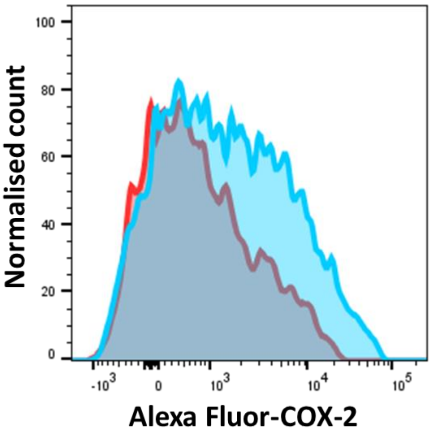

Figure S26. Representative histograms displaying the green fluorescence emitted by anti-COX-2 Alexa Fluor 488 nm antibody-stained HMLER-shEcad cells without (red) and with (blue) LPS (2.5 μM) for 24 h, followed by 48 h in media.

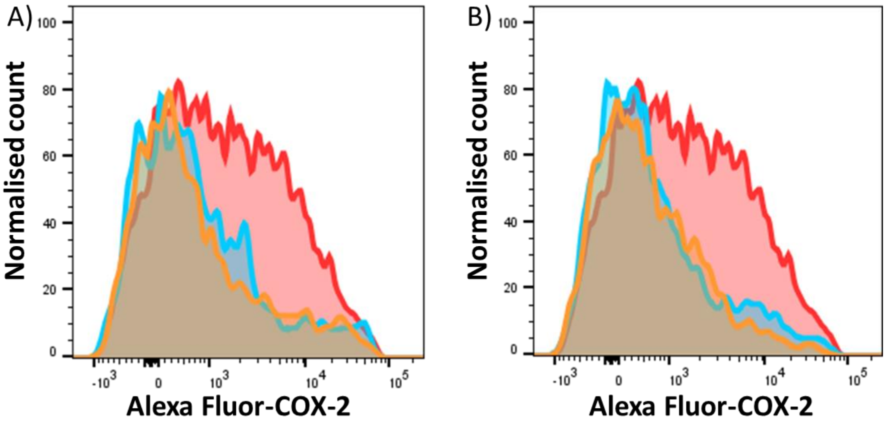

Figure S27. (A) Representative histograms displaying the green fluorescence emitted by anti-COX-2 Alexa Fluor 488 nm antibody-stained HMLER-shEcad cells treated with LPS (2.5 μM) for 24 h, followed by 48 h in media (red) or media containing **1** (IC_{50} value, blue) or **1** (2 × IC_{50} value, orange). (B) Representative histograms displaying the green fluorescence emitted by anti-COX-2 Alexa Fluor 488 nm antibody-stained HMLER-shEcad cells treated with LPS (2.5 μM) for 24 h, followed by 48 h in media (red) or media containing flufenamic acid (20 μM, blue) or flufenamic acid (40 μM, orange).

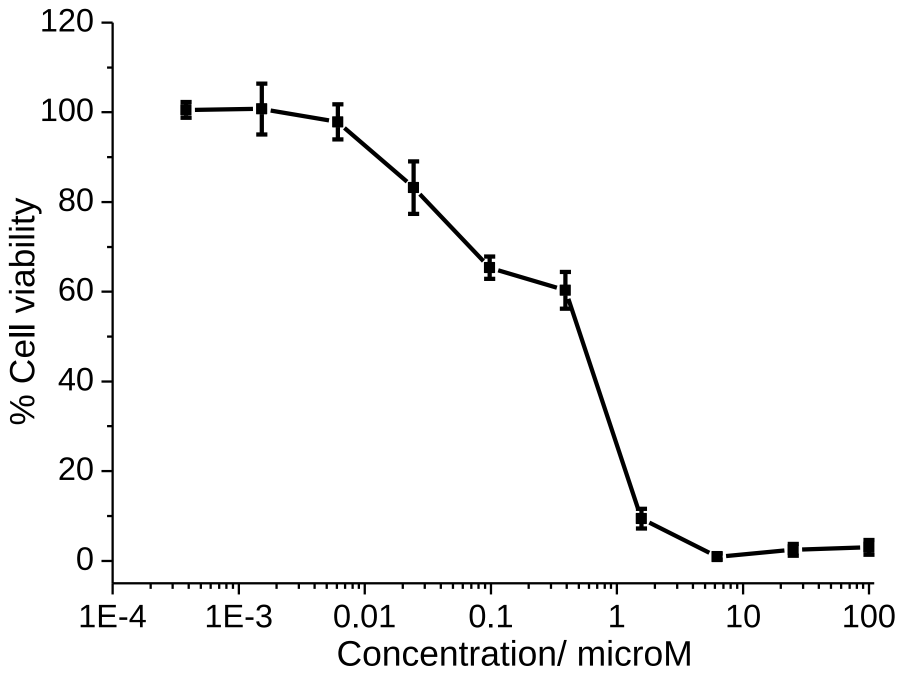

Figure S28. Representative dose-response curves for the treatment of LPS pre-treated (2.5 μM for 24 h) HMLER-shEcad cells with **1** after 72 h incubation. Error bars represent standard deviations.

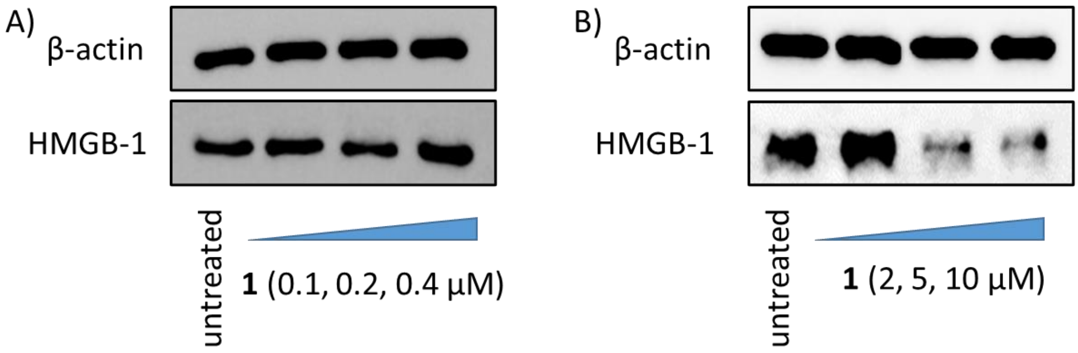

Figure S29. Immunoblotting analysis of high mobility group box 1 (HMGB-1). Protein expression in HMLER-shEcad cells following treatment with (A) **1** (0.1-0.4 μM for 72 h) or (B) **1** (2-10 μM for 24 h).

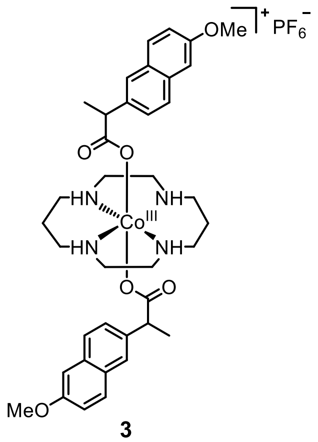

3

Figure S30. Chemical structure of the previously reported cobalt(III)-cyclam complex bearing two naproxen moieties, **3**.

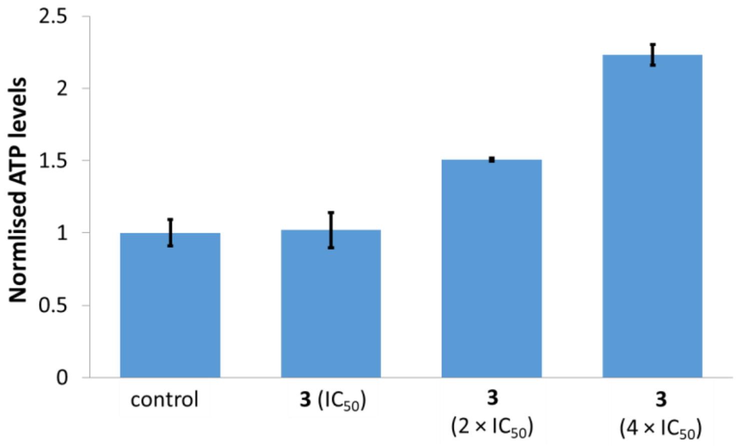

Figure S31. Normalised extracellular ATP released from HMLER-shEcad cells untreated and treated with **3** (IC_{50} value, 2 × IC_{50} value, and 4 × IC_{50} value for 24 h). Error bars represent standard deviations.

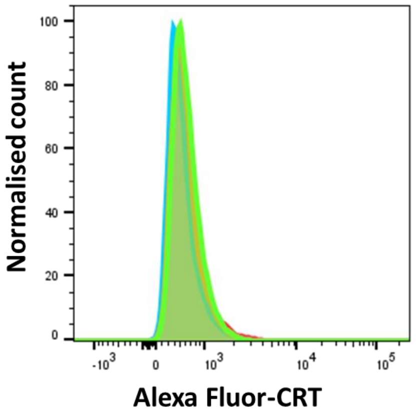

Figure S32. Representative histograms displaying the green fluorescence emitted by anti-CRT Alexa Fluor 488 nm antibody-stained HMLER-shEcad cells untreated (red), and treated with **3** (IC_{50} value for 24 h) (blue) or **3** (2 × IC_{50} value for 24 h) (orange) or **3** (4 × IC_{50} value for 24 h) (green).

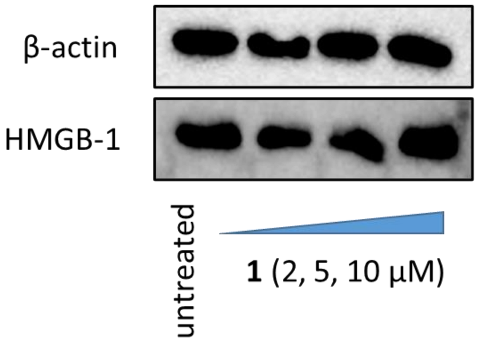

Figure S33. Immunoblotting analysis of high mobility group box 1 (HMGB-1). Protein expression in HMLER-shEcad cells following treatment with **3** (2-10 μM for 24 h).

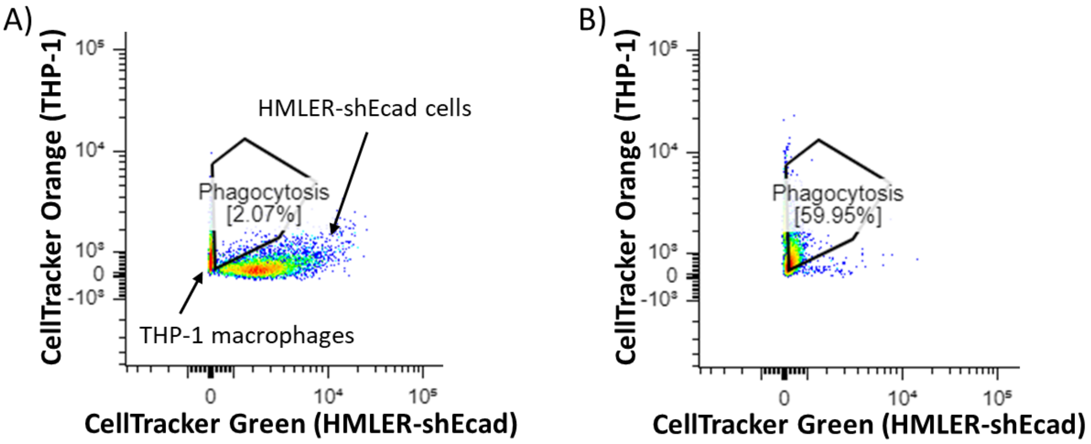

Figure S34. Representative two-dimensional scatter plots of CellTracker Green-stained HMLER-shEcad cells (A) untreated and (B) treated with cisplatin (150 μM for 24 h) and thapsigargin (7 μM for 24 h) and then co-cultured with CellTracker Orange-stained THP-1 macrophages for 2 h. The population of HMLER-shEcad cells phagocytosed by THP-1 macrophages is indicated.

References

[1] B. Bosnich, C. K. Poon, M. L. Tobe, *Inorg. Chem.* **1965**, *4*, 1102-1108.

[2] G. Sheldrick, *University of Göttingen, Germany* **1996**.

[3] CrysAlisPro, Agilent Technologies, Version 1.171.35.11. Multi-scans absorption correction with SCALE3 ABSPACK scaling algorithm.

[4] G. M. Sheldrick, *Acta Crystallogr. Sect. C* **2015**, *71*, 3-8.
